# Cellular ESCRT components are recruited to regulate the endocytic trafficking and RNA replication compartment assembly during classical swine fever virus infection

**DOI:** 10.1371/journal.ppat.1010294

**Published:** 2022-02-04

**Authors:** Chun-chun Liu, Ya-yun Liu, Jiang-fei Zhou, Xi Chen, Huan Chen, Jia-huan Hu, Jing Chen, Jin Zhang, Rui-cong Sun, Jian-chao Wei, Yun Young Go, Eiji Morita, Bin Zhou

**Affiliations:** 1 MOE Joint International Research Laboratory of Animal Health and Food Safety, College of Veterinary Medicine, Nanjing Agricultural University, Nanjing, China; 2 Shanghai Veterinary Research Institute, Chinese Academy of Agricultural Sciences, Shanghai, China; 3 Department of Infectious Diseases and Public Health, City University of Hong Kong, Hong Kong SAR, China; 4 Department of Biochemistry and Molecular Biology, Faculty of Agriculture and Life Science, Hirosaki University, Hirosaki, Japan; SPAIN

## Abstract

As the important molecular machinery for membrane protein sorting in eukaryotic cells, the endosomal sorting and transport complexes (ESCRT-0/I/II/III and VPS4) usually participate in various replication stages of enveloped viruses, such as endocytosis and budding. The main subunit of ESCRT-I, Tsg101, has been previously revealed to play a role in the entry and replication of classical swine fever virus (CSFV). However, the effect of the whole ESCRT machinery during CSFV infection has not yet been well defined. Here, we systematically determine the effects of subunits of ESCRT on entry, replication, and budding of CSFV by genetic analysis. We show that EAP20 (VPS25) (ESCRT-II), CHMP4B and CHMP7 (ESCRT-III) regulate CSFV entry and assist vesicles in transporting CSFV from Clathrin, early endosomes, late endosomes to lysosomes. Importantly, we first demonstrate that HRS (ESCRT-0), VPS28 (ESCRT-I), VPS25 (ESCRT-II) and adaptor protein ALIX play important roles in the formation of virus replication complexes (VRC) together with CHMP2B/4B/7 (ESCRT-III), and VPS4A. Further analyses reveal these subunits interact with CSFV nonstructural proteins (NS) and locate in the endoplasmic reticulum, but not Golgi, suggesting the role of ESCRT in regulating VRC assembly. In addition, we demonstrate that VPS4A is close to lipid droplets (LDs), indicating the importance of lipid metabolism in the formation of VRC and nucleic acid production. Altogether, we draw a new picture of cellular ESCRT machinery in CSFV entry and VRC formation, which could provide alternative strategies for preventing and controlling the diseases caused by CSFV or other Pestivirus.

## Author summary

ESCRT machinery can be responsible for virus budding and participate in regulating virus entry. However, little has been reported on its effects on VRC formation. Here, we uncover the novel roles of ESCRT-III and VPS4A in VRC assembly and update the additional subunits involved in the intracellular trafficking of CSFV. These data indicate that the ESCRT machinery promotes CSFV replication by forming VRC, which making it become nuclease-insensitive to avoid the recognition by the host antiviral surveillance system and the destruction of the viral RNA. Furthermore, we first demonstrate that the roles of ESCRT components in the formation of VRC in swine Pestivirus. Our findings highlight the growing evidence of diverse interactions between ESCRT subunits and viral factors of Flaviviridae family, and provide alternative strategies for preventing and controlling the diseases caused by CSFV or other Pestivirus.

## Introduction

A virus composed of genetic material with a protein shell usually exploits specific viral transport protein on the host cell membrane for endocytosis and releases a large amount of viral genetic materials for replication [1]. The virus requires cellular factors to replicate its genome and usurps cell function to serve itself. Both enveloped and non-enveloped viruses depend on the host endocytic pathways for entry and advance this processes through different ways, allowing themselves to move from the cell periphery to the perinuclear space [2–4]. Continually, viruses take advantages of host factors for replication and spread [5]. To date, a map of the interaction between host cells and viral proteins has been drawn through proteomics and transcriptomics studies of infected cells, and systematic RNA interference (RNAi) screening has also been developed to identify host factors involved in viral replication. Most members of the Flaviviridae family are positive-sense RNA (+RNA) viruses, which greatly change the structure of host cells, serving as a platform for the replication and assembly of new virions [6,7]. Hereafter, the interaction between viral and host factors leads to the formation of replication compartments, such as the virus replication complex (VRC) [8,9].

ESCRT machinery is important for the endosomal/multivesicular body (MVB) protein-sorting pathway in eukaryotic cells [10,11], which regulates plasma membrane proteins via endocytosis [12] and sorts newly synthesized membrane proteins from trans-Golgi vesicles to the endosomes, lysosomes, or the plasma membranes [13]. The ESCRT proteins are involved in membrane invagination and vesicle formation in the MVB pathway [14]. It has been shown that the ESCRT system is composed of more than 20 proteins [15,16]. The ESCRT system can be divided into five complexes, ESCRT-0, I, II, III, VPS4 (vacuum protein sorting associated protein 4) and some auxiliary proteins, such as ALIX [ALG-2 (apoptosis linked gene 2)—interacting protein X], etc. Enveloped retroviruses (such as HIV) and +RNA viruses (such as filo-, arena-, radon- and paramyxoviruses) redirect cellular ESCRT proteins to the plasma membrane, leading to budding and fission of the viral particles from infected cells [17,18].

Classical swine fever virus (CSFV) is an enveloped virus of icosahedral symmetry with a diameter between 40–60 nm. The viral genome is a single-stranded +RNA of approximately 12.3 kb with a structure of a single open reading frame (ORF) surrounded by two untranslated regions (UTRs). The uncapped 5′-UTR carries an internal ribosome entry site (IRES), and the 3′-UTR is uridine-rich. The ORF encodes a polyprotein that is cleaved into four structural (capsid protein C, envelope glycoproteins Erns, E1, and E2) and eight nonstructural proteins (Npro, p7, NS2, NS3, NS4A, NS4B, NS5A and NS5B) [19,20]. Though cell entry is the critical step in the virus replication cycle, the host receptor of CSFV has not been identified. A recent study has suggested that the laminin receptor plays an integral role in virus entry, and Erns is required for cell binding [21,22]. Our previous studies demonstrated that CSFV enters host cells through Clathrin- or Caveolin-1 mediated endocytosis [23,24], which depends on the type of cell line and requires cholesterol and low pH for productive infection [25]. Further, CSFV is transported from early endosomes (Rab5) to late endosomes (Rab7) or circulating endosomes (Rab11) and finally to lysosomes (LAMP-1) to complete the uncoating process [23,24]. Moreover, our recent study showed that CSFV glycoprotein E2 co-localize with Tsg101 from 15 min post endocytosis, suggesting an important role of ESCRT in CSFV entry [26].

Interestingly, it was found that CSFV proliferation is significantly inhibited after knocking down endogenous Tsg101. Then, it was further identified that Tsg101 protein locates in the endoplasmic reticulum (ER) and interacts with NS4B/5B proteins and dsRNA to form the viral replication complex (VRC) [26]. Given the function of the ESCRT system in regulating endocytosis maturation and transport [15,27], the purpose of this study is to identify the key components of the ESCRT system involved in CSFV entry, replication, or budding. Here, we first used systematic siRNA screening to identify the ESCRT subunits that participate in CSFV infection. VPS25, CHMP4B and CHMP7 were found to be recruited into the area of the post-entry endosomal compartments to promote CSFV endocytosis. Importantly, HRS, VPS28, VPS25, CHMP2B/4B/7, VPS4A, and ALIX form viral replication complex (VRC) in the ER region by interacting with CSFV nonstructural proteins with the help of dsRNA. Taken together, CSFV infection makes full use of ESCRT components to complete the process of entry and replication.

## Result

### ESCRT is involved in CSFV infection

**Fig 1:**
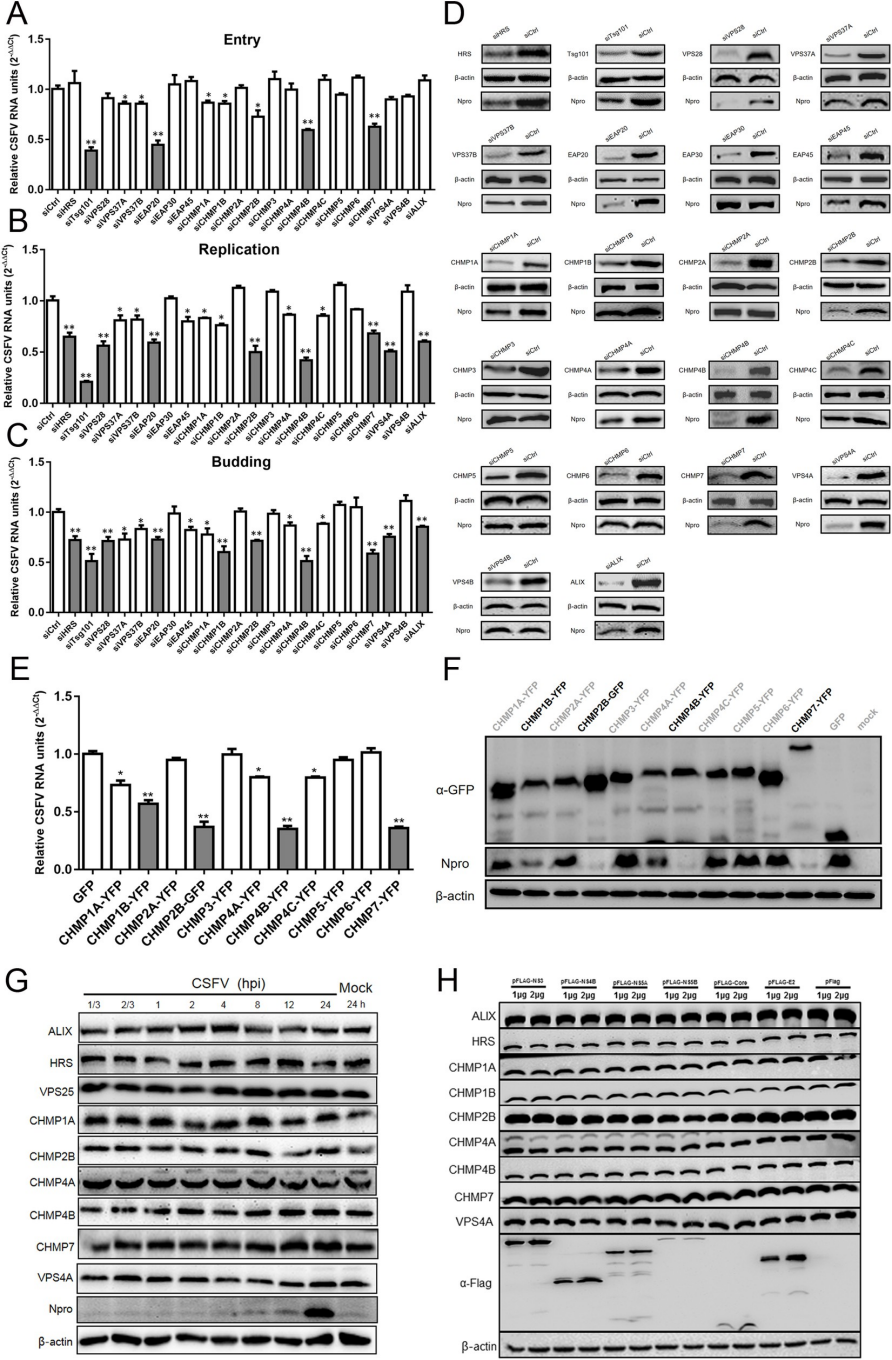
ESCRT is involved in CSFV infection. (A) PK-15 cells were transfected with siESCRTs or siCtrl and then inoculated with CSFV (MOI = 10), and the cells were harvested for RT-qPCR at 1 hpi. These data are presented as the mean + SD of data from three independent experiments. *, P< 0.05; **, P <0.01. (B) PK-15 cells were transfected with siESCRTs or siCtrl and then inoculated with CSFV (MOI = 0.1), then harvested the whole cell cultures at 24 hpi for RT-qPCR. These data are presented as the mean + SD of data from three independent experiments. *, P< 0.05; **, P <0.01. (C) PK-15 cells were transfected with siESCRTs or siCtrl and then inoculated with CSFV (MOI = 0.1), at 24 hpi, the cell supernatant were harvested and used for infected new PK-15 cells, and harvested the new cells at 24 hpi for RT-qPCR. These data are presented as the mean + SD of data from three independent experiments. *, P< 0.05; **, P <0.01. (D) PK-15 cells were treated as described in panel B. At 24 hpi, the cells were harvested and subjected to Western blotting by using the indicated antibodies as follows: rabbit anti-ESCRTs antibody, rabbit anti-Npro antibody, or mouse anti-β-actin antibody. These data are representative of three independent experiments. (E and F) PK-15 cells were transfected YFP-tagged ESCRT-III or vector plasmid and then inoculated with CSFV (MOI = 0.1). At 24 hpi, the cell culture was harvested, respectively, for RT-qPCR and Western blotting described above. These data are presented as the mean + SD of data from three independent experiments. *, P< 0.05; **, P <0.01. (G) PK-15 cells were infected with CSFV (MOI = 1) for indicated time points, then cells were harvested and subjected to Western blotting by using the indicated antibodies against ALIX, HRS, VPS25, CHMP1A, CHMP2B, CHMP4A, CHMP4B, CHMP7, VPS4A, Npro and β-actin. These data are representative of three independent experiments. (H) PK-15 cells were transfected doses of indicated plasmids (pFlag-NS3, -NS4B, -NS5A, -NS5B, -Core, -E2) or vector for 48 hpt, then harvested and subjected to Western blotting by using rabbit anti-ESCRTs antibody or mouse anti-Flag antibody, along with β-actin as a loading control. These data are representative of three independent experiments.

To investigate whether the ESCRT subunits play a role in the life cycle of CSFV, we measured the different stages of CSFV infection in cells by transfection with the siRNA targeting each ESCRT subunit. The cytotoxic effects of all targeted siRNAs on PK-15 cells were evaluated by the CCK8 assay, and no cytotoxicity was observed in cells treated with the indicated concentrations of siRNA duplexes (S1A Fig). As shown in Figs 1A and S1B, compared with the control group, the levels of viral mRNA were significantly reduced after knockdown of endogenous Tsg101, EAP20 (VPS25), CHMP4B, and CHMP7 in the early entry stage (1h post-infection), indicating that these ESCRT complex subunits play important roles in the early entry stage of CSFV. The depletion of VPS37A, VPS37B, CHMP1A, CHMP1B and CHMP2B also slightly affected the amount of viral RNA, suggesting that these ESCRT complex subunits may have limited roles in the viral entry step. Furthermore, at 24 h post-infection, the levels of viral mRNA were significantly downregulated after knockdown of endogenous HRS, Tsg101, VPS28, EAP20, CHMP2B, CHMP4B, CHMP7, VPS4A and ALIX, also VPS37A, VPS37B, EAP45, CHMP1A, CHMP1B, CHMP4A and CHMP4C slightly reduced the amount of viral RNA (Figs 1B and S1C), compared with the control treatment. These results indicate that at least these ESCRT subunits have a specific role in the virus replication. By the way, we also noted that HRS, Tsg101, VPS28, EAP20, CHMP1B, CHMP2B, CHMP4B, CHMP7, VPS4A and ALIX had an influence on the stage of viral budding (Figs 1C and S1D). We further confirmed these results by western blotting. Consistent with the RT-qPCR data, the protein levels of CSFV Npro were significantly decreased with the depletion of the essential ESCRT subunit proteins described above (Figs 1D, S1E and S1F). Meanwhile, an indirect immunofluorescence experiment was also performed. There was a reduction in the fluorescence signals of CSFV E2 proteins upon endogenous CHMP2B/4B/7 or VPS4A knockdown (S1G Fig). As illustrated in Figs 1B, 1D, S1C, S1E, S1F and S1G, subunits of ESCRT-III participated in the CSFV replication. It has been demonstrated that dominant-negative (DN) forms of ESCRT-III proteins efficiently inhibit the hepatitis C virus (HCV) by blocking the ESCRT pathway [28]. Here, we observed a similar inhibitory effect against CSFV replication after overexpression of the YFP-fused ESCRT-III subunit proteins. As shown in Fig 1E and 1F, CSFV replication was significantly reduced after overexpression of the DN constructs of CHMP1B, CHMP2B, CHMP4B and CHMP7, and slightly decreased after overexpression of the DN constructs of CHMP1A, CHMP4A and CHMP4C, compared with the vector. Conversely, we determined whether CSFV infection affects the expression of endogenous ESCRT subunits. As shown in Fig 1G, the level of endogenous ESCRT subunit proteins did not change significantly after CSFV infection. Furthermore, the endogenous ESCRT subunits expression levels almost kept the same after the cells were transfected with different doses of CSFV structural or nonstructural proteins (Fig 1H). These results support that the subunits of the ESCRT protein are involved in different stages of CSFV infection.

### ESCRT subunits participate in CSFV endocytosis

**Fig 2:**
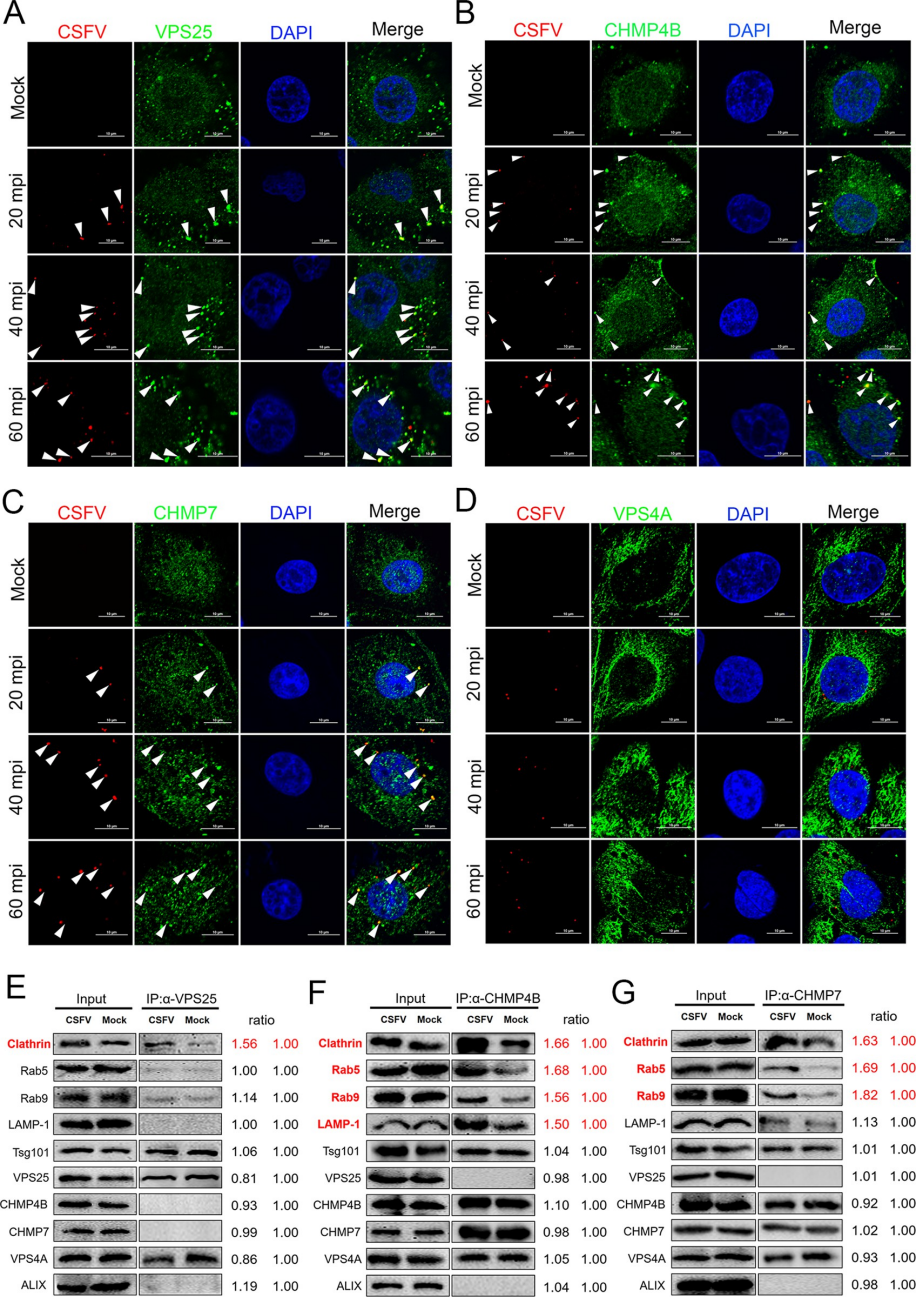
ESCRTs machinery participates in CSFV endocytosis. (A-D) PK-15 cells were infected with CSFV (MOI = 10) at 4°C for 1 hpi, then shifted to 37°C for indicated time points, respectively. After fixed and subjected to immunofluorescent by using mouse anti-CSFV E2 monoclonal antibody (WH303) and rabbit anti-VPS25 (A); -CHMP4B (B); -CHMP7 (C); -VPS4A (D) antibody. The nuclei were stained with DAPI. The white arrow indicates the co-localized protein. Bars = 10 μm. These data are representative of three independent experiments. (E-G) PK-15 cells were infected with CSFV (MOI = 10) or not for 6 hpi, then harvested for immunoprecipitation by using mouse anti-VPS25 antibody (E) or rabbit anti-CHMP4B (F); -CHMP7 antibody (G), and the whole-cell lysates were subjected to Western blotting by using the antibodies against Clathrin, Rab5, Rab9, LAMP-1, Tsg101, VPS25, CHMP4B, CHMP7, VPS4A and ALIX. These data are representative of three independent experiments.

To study whether VPS25, CHMP4B and CHMP7 are involved in the early stage of CSFV infection, PK-15 cells were infected with CSFV, then stained with CSFV virions (E2 protein) and endogenous ESCRT subunits, respectively. As shown in Fig 2A to 2D, the viral particles (E2 protein) co-localized with endogenous VPS25, CHMP4B, or CHMP7, but not with VPS4A (the negative control). These results suggest that VPS25, CHMP4B and CHMP7 are recruited to the entry site of CSFV particles. Pearson’s correlation analysis also confirmed these colocalizations (S2A Fig). In addition, to detect whether other associated factors are accumulated at the entry sites together with VPS25, CHMP4B or CHMP7, we performed a Co-IP assay with mock- or CSFV-infected cells at 6 hpi, using specific antibodies against VPS25, CHMP4B or CHMP7. We found that the interaction between VPS25 and Clathrin was significantly enhanced after CSFV infection, compared with mock (Figs 2E and S2B). In addition, CHMP4B’s interactions with Clathrin, LAMP-1, Rab5 and Rab9 were increased after CSFV infection (Figs 2F and S2C). As for CHMP7, the interactions with Clathrin, Rab5 and Rab9 were significantly enhanced upon CSFV infection (Figs 2G and S2D). Furthermore, an immunofluorescent microscopy assay was performed to confirm the above-mentioned interactions of VPS25, CHMP4B, and CHMP7. As shown in S3A and S3B Fig, the endogenous VPS25 was significantly co-localized with Clathrin protein but not with other proteins after CSFV infection. As expected, the endogenous CHMP4B was significantly co-localized with Clathrin, Rab5, Rab9, and LAMP-1 after CSFV infection (S4A and S4B Fig). And the endogenous CHMP7 was co-localized with Clathrin, Rab5 and Rab9 after CSFV infection, but not LAMP-1 (S5A and S5B Fig). These results indicate that VPS25, CHMP4B and CHMP7 regulate the entry step of CSFV by recruiting Clathrin and different endosomes.

### ESCRT participates in the formation of the CSFV replication complex

**Fig 3:**
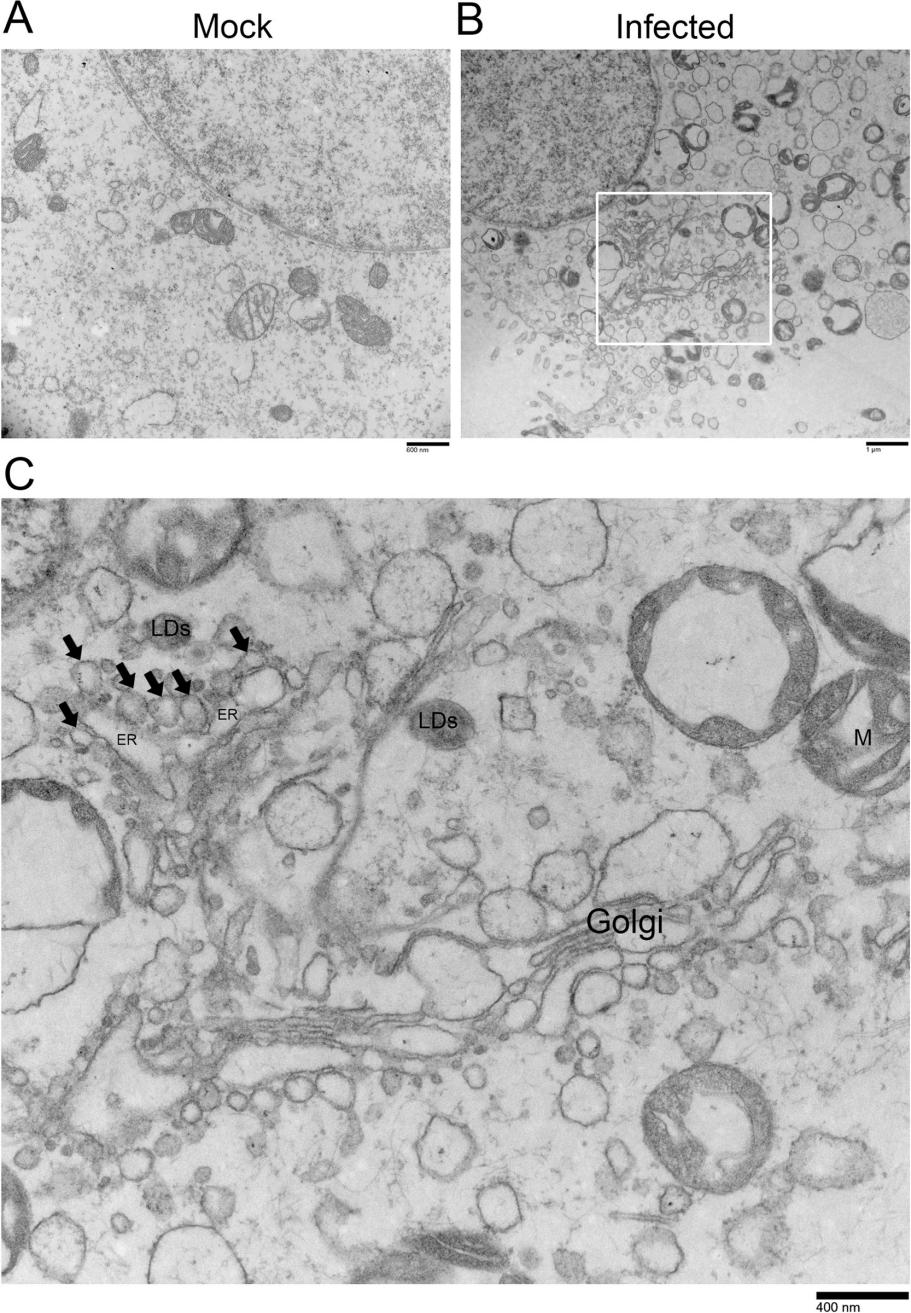
Visualization of the location of the CSFV replication complex. (A and B) PK-15 cells were infected with CSFV (MOI = 10) or not for 24 hpi, then harvested and subjected to electron microscopy. (C) This picture is the field of view enlarged by the white box in panel B, the black arrow indicated the virus replication complex (VRC). Additionally, LDs means Lipid droplets, M means mitochondrion. These data are representative of three independent experiments.

It has been demonstrated that CSFV creates a specific compartment called CSFV replication complex (VRC) for efficient viral genome replication [26]. We employed electron microscopy to examine the location and formation of VRC. We observed that many hemispherical or spherical vesicles were produced upon virus infection compared with the mock control (Fig 3A and 3B). Interestingly, these VRC vesicles were associated with ER [29], where intimal depression was induced, as well as dsRNA and associated proteins during virus replication (Fig 3C).

**Fig 4:**
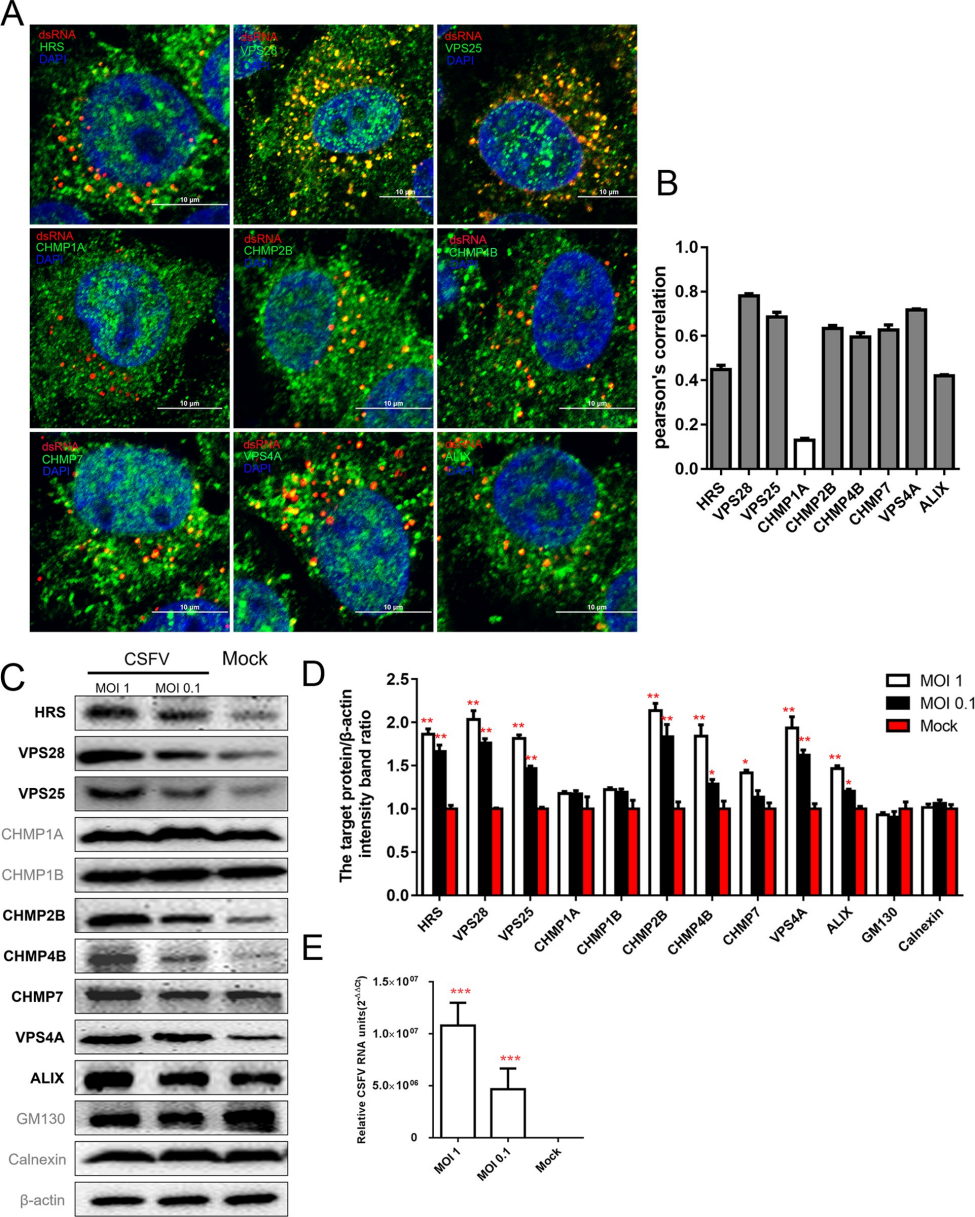
ESCRT participates in the formation of the CSFV replication complex. (A) PK-15 cells were inoculated with CSFV (MOI = 1) for 24 hpi, then fixed and subjected to immunofluorescent by using mouse anti-dsRNA antibody (red) and rabbit anti-ESCRTs antibody (green). The nuclei were stained with DAPI. Bars = 10 μm. These data are representative of three independent experiments. (B) The colocalization analysis was expressed as Pearson’s correlation coefficient, respectively. Results are represented as the mean + SD of data from three independent experiments. (C) PK-15 cells were infected with CSFV at different MOI for 24 hpi and extracted viral replication complex related proteins. The extraction was subjected to Western blotting by using rabbit anti-ESCRTs antibody, rabbit anti-GM130 antibody, or rabbit anti-Calnexin antibody, along with mouse anti-β-actin antibody as control. These data are representative of three independent experiments. (D) Western blotting results were analyzed of grayscale analysis through image J software, respectively. The white column indicates that the MOI is 1, the black column indicates that the MOI is 0.1 and the red column represents mock. These data are presented as the mean + SD of data from three independent experiments. *, P< 0.05; **, P <0.01. (E) PK-15 cells were treated as described in panel C, then harvested and subjected to RT-qPCR. These data are presented as the mean + SD of data from three independent experiments. ***, P <0.001.

To determine whether the ESCRT subunits (HRS, VPS28, VPS25, CHMP2B, CHMP4B, CHMP7, VPS4A and ALIX) influence CSFV genome replication and are involved in the formation of VRC, we tested the co-localizations of these ESCRT subunits with double-stranded RNA (dsRNA), known as intermediate products of CSFV genome replication. As shown in Fig 4A and 4B, dsRNA signals were observed to co-localize with endogenous HRS, VPS28, VPS25, CHMP2B, CHMP4B, CHMP7, VPS4A and ALIX, but not with CHMP1A (the internal negative control). Next, we performed biochemical assays to extract and purify VRC from the PK-15 cells infected with CSFV at different MOIs. As shown in Fig 4C, 4D and 4E, the protein levels of these ESCRT subunits (HRS, VPS28, VPS25, CHMP2B, CHMP4B, CHMP7, VPS4A and ALIX) in the VRC were significantly increased by the viral infection in a dose-dependent manner. These results indicate that these ESCRT subunits are recruited to the viral genome replication site during viral infection, and play important roles in the VRC formation and virus replication.

### CSFV proteins interact with main subunits of the ESCRT system

To study the viral components involved in the ESCRT-mediated process, we performed confocal fluorescence microscopy and immunoprecipitation to detect the interactions between viral proteins (Core, E2, NS3, NS4B, NS5A and NS5B) and above-mentioned ESCRT subunits (HRS, VPS28, VPS25, CHMP2B, CHMP4B, CHMP7, VPS4A and ALIX).

**Fig 5:**
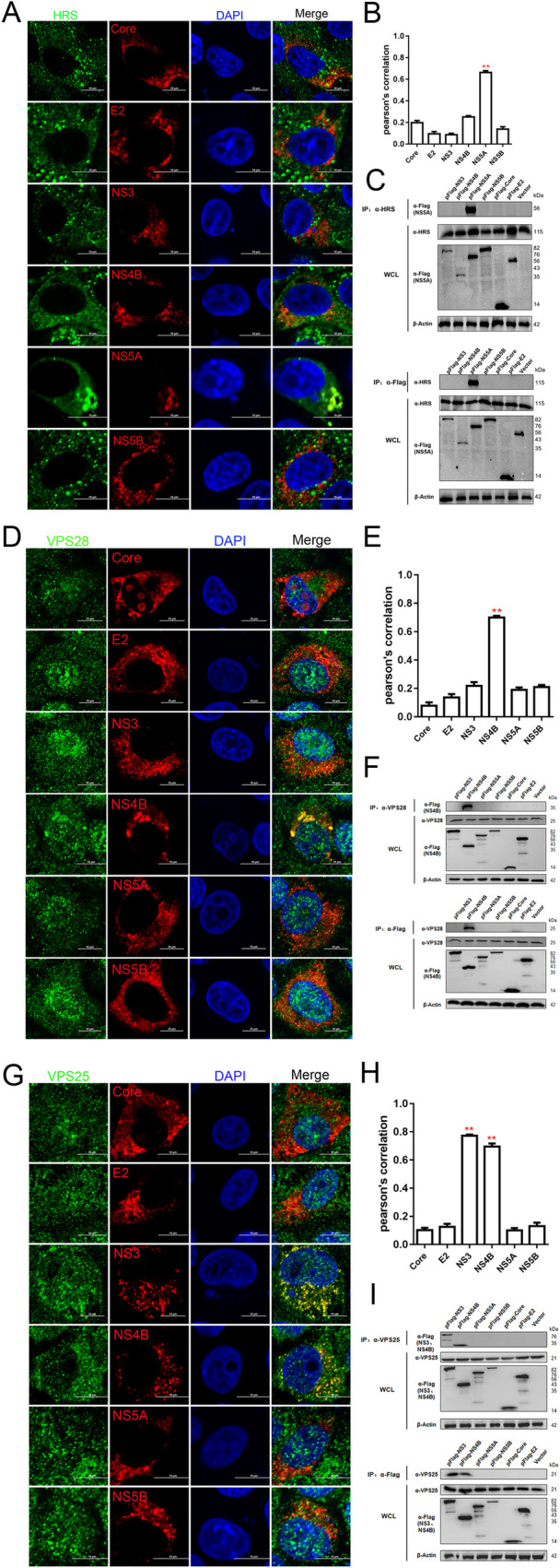
Interaction between major subunits of the ESCRT-0/I/II system and CSFV proteins. (A, D and G) PK-15 cells were transfected with indicated plasmids (pFlag-Core, -E2, -NS3, -NS4B, -NS5A, -NS5B) for 48 hpt, then fixed and subjected to immunofluorescent by using rabbit anti-HRS (A); -VPS28 (D); -VPS25 (G) antibody (green) and mouse anti-Flag antibody (red). The nuclei were stained with DAPI. Bars = 10 μm. These data are representative of three independent experiments. (B, E and H) The colocalization analysis was expressed as Pearson’s correlation coefficient, respectively. Results are represented as the mean + SD of data from three independent experiments. **, P < 0.01. (C, F and I) HEK-293T cells were transfected with indicated plasmids (pFlag-NS3, -NS4B, -NS5A, -NS5B, -Core, -E2) or vector for 48 hpt, then harvested for immunoprecipitation by using mouse anti-Flag antibody or mouse anti-HRS (C); -VPS28 (F); -VPS25 (I) antibody, and whole-cell lysates were subjected to Western blotting by using rabbit anti-HRS/VPS28/VPS25 antibody or mouse anti-Flag antibody, along with β-actin as a loading control. These data are representative of three independent experiments.

HRS is a subunit of ESCRT-0 and working on transferring cargo protein to the ESCRT-I complex [30,31]. Immunofluorescent studies showed that both endogenous HRS and exogenous HRS were co-localized with NS5A, but not with Core, E2, NS3, NS4B and NS5B (Figs 5A, 5B, S6A and S6B). Only the interaction between NS5A and endogenous HRS was observed from the immunoprecipitation assay, consistent with the immunofluorescent studies (Fig 5C). VPS28 is a subunit of ESCRT-I and can interact with ESCRT-II complex to connect the ESCRT-0 and ESCRT-II complexes together [32,33]. As shown in Fig 5D, 5E and 5F, VPS28 was detected to co-localize and interact with NS4B, but not with Core, E2, NS3, NS5A and NS5B. In addition, VPS25, the subunit of ESCRT-II, was found to co-localize with not only NS3 but also NS4B (Fig 5G and 5H), which was also confirmed by the immunoprecipitation experiments (Fig 5I).

**Fig 6:**
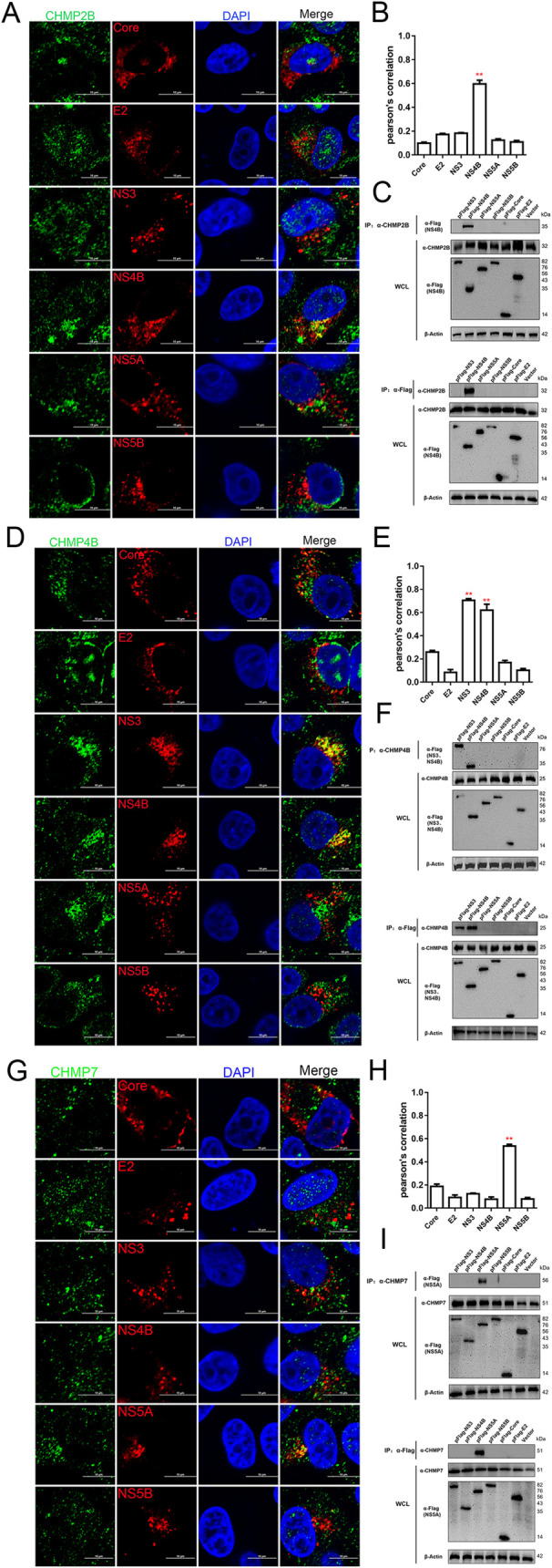
Interaction between major subunits of the ESCRT-III system and CSFV proteins. (A, D and G) PK-15 cells were transfected with indicated plasmids (pFlag-Core, -E2, -NS3, -NS4B, -NS5A, -NS5B) for 48 hpt, then fixed and subjected to immunofluorescent by using rabbit anti-CHMP2B (A); -CHMP4B (D); -CHMP7 (G) antibody (green) and mouse anti-Flag antibody (red). The nuclei were stained with DAPI. Bars = 10 μm. These data are representative of three independent experiments. (B, E and H) The colocalization analysis was expressed as Pearson’s correlation coefficient, respectively. Results are represented as the mean + SD of data from three independent experiments. **, P < 0.01. (C, F and I) HEK-293T cells were transfected with indicated plasmids (pFlag-NS3, -NS4B, -NS5A, -NS5B, -Core, -E2) or vector for 48 hpt, then harvested for immunoprecipitation by using mouse anti-Flag antibody or rabbit anti-CHMP2B (C); -CHMP4B (F); -CHMP7 (I) antibody, and whole-cell lysates were subjected to Western blotting by using rabbit anti-CHMP2B/CHMP4B/CHMP7 antibody or mouse anti-Flag antibody, along with β-actin as a loading control. These data are representative of three independent experiments.

CHMP2B, CHMP4B and CHMP7 are subunits of the ESCRT-III complex. The immunofluorescent studies showed that both endogenous CHMP2B and exogenous CHMP2B were co-localized with NS4B, but not with Core, E2, NS3, NS5A and NS5B (Figs 6A, 6B, S7A and S7B). Consistently, NS4B interacted with CHMP2B, as we have seen in the immunoprecipitation experiments (Fig 6C). CHMP4B was co-localized and interacted with NS3 and NS4B (Fig 6D, 6E and 6F). CHMP7 was found to only co-localize and interact with NS5A (Fig 6G, 6H and 6I).

**Fig 7:**
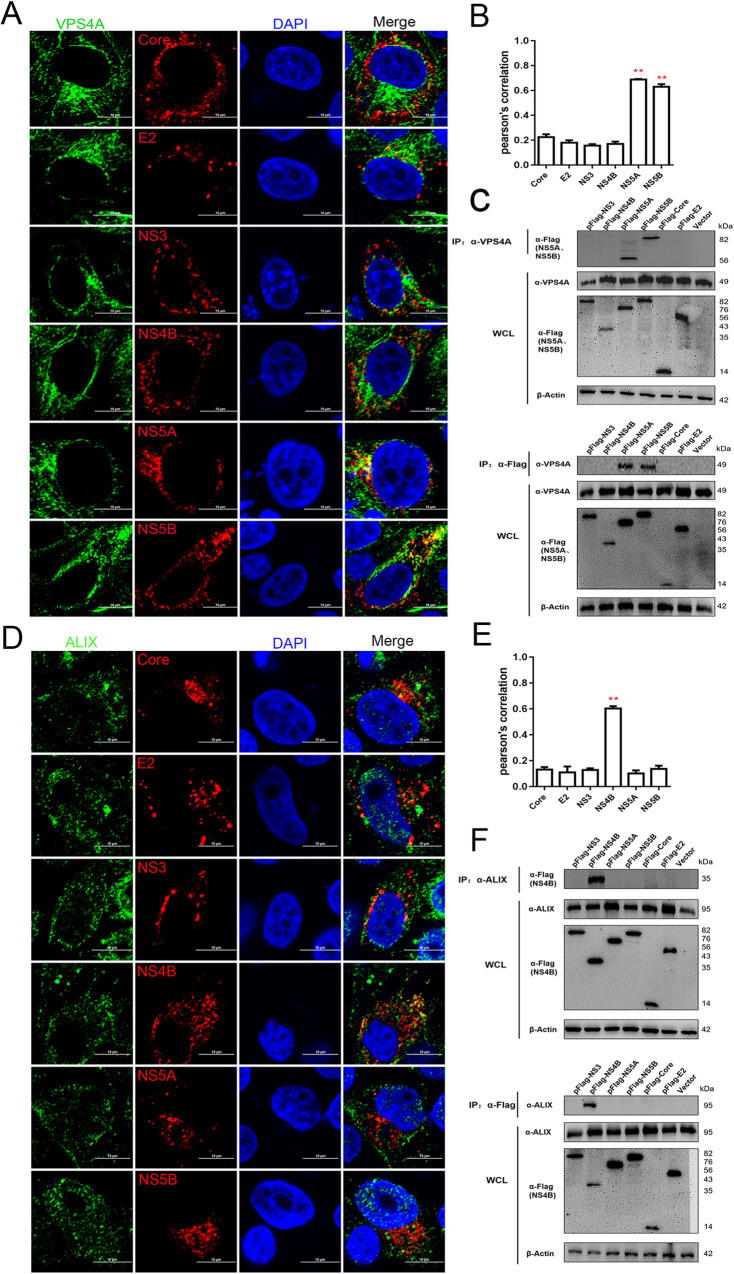
Interaction between endogenous VPS4A/ALIX proteins and CSFV proteins. (A and D) PK-15 cells were transfected with indicated plasmids (pFlag-Core, -E2, -NS3, -NS4B, -NS5A, -NS5B) for 48 hpt, then fixed and subjected to immunofluorescent by using rabbit anti-VPS4A (A); -ALIX (D) antibody (green) and mouse anti-Flag antibody (red). The nuclei were stained with DAPI. Bars = 10 μm. These data are representative of three independent experiments. (B and E) The colocalization analysis was expressed as Pearson’s correlation coefficient, respectively. Results are represented as the mean + SD of data from three independent experiments. **, P < 0.01. (C and F) HEK-293T cells were transfected with indicated plasmids (pFlag-NS3, -NS4B, -NS5A, -NS5B, -Core, -E2) or vector for 48 hpt, then harvested for immunoprecipitation by using mouse anti-Flag antibody or mouse anti-VPS4A antibody (C); rabbit anti-ALIX antibody (F), and whole-cell lysates were subjected to Western blotting by using rabbit anti-VPS4A/ALIX antibody or mouse anti-Flag antibody, along with β-actin as a loading control. These data are representative of three independent experiments.

VPS4 protein forms an independent complex from ESCRT-0 to ESCRT-III, which plays a role in depolymerizing the ESCRT-III polymers at the expense of ATP hydrolysis, leading to the disassembly of membrane-bound ESCRT-III complex for recycling use [34,35]. The immunofluorescent studies show that both endogenous VPS4A and exogenous VPS4A were co-localized with not only NS5A but also NS5B (Figs 7A, 7B, S8A and S8B). The immunoprecipitation experiments also confirmed that VPS4A was co-precipitated with NS5A and NS5B (Fig 7C). ALIX can combine with both ESCRT-I and ESCRT-III (CHMP4 proteins) and link to upstream and downstream ESCRT complex [36,37]. The results showed that ALIX was co-localized with NS4B (Fig 7D and 7E), and only Flag-tagged NS4B co-precipitated with ALIX (Fig 7F). Taken together, these results suggest that each ESCRT subunit has single or multiple specific partners for the recruitment to the site of viral replication.

### ESCRT subunits are associated with the endoplasmic reticulum (ER)

**Fig 8:**
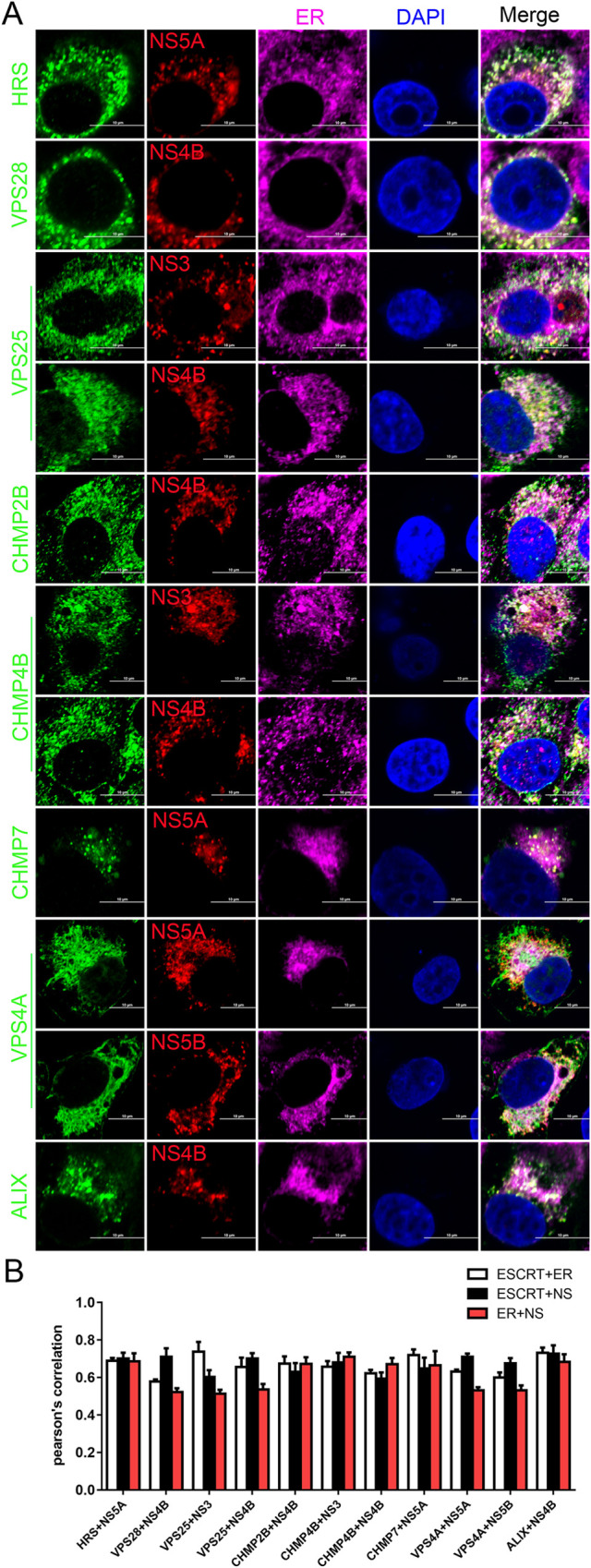
ESCRT subunits are involved in the replication complex of CSFV. (A) PK-15 cells were transfected with indicated plasmids (pFlag-NS3, -NS4B, -NS5A, -NS5B) for 48 hpt, then fixed and subjected to immunofluorescent by using mouse anti-HRS/VPS28/VPS25/CHMP7/VPS4A/ALIX antibody (green), goat anti-Flag antibody (red) and rabbit anti-Calnexin antibody (purple); or rabbit anti-CHMP2B/CHMP4B antibody (green), goat anti-Flag antibody (red) and mouse anti-Calnexin antibody (purple). The nuclei were stained with DAPI. Bars = 10 μm. These data are representative of three independent experiments. (B) The colocalization coefficient of ESCRTs, NS (nonstructural proteins) and ER was expressed as Pearson’s correlation coefficient. The white column indicates the co-localization of the ESCRT subunits and ER, the black column indicates the co-localization of the ESCRT subunits and nonstructural proteins, and the red column indicates the co-localization of the ER and nonstructural proteins. Results are represented as the mean + SD of data from three independent experiments.

To investigate the subcellular distributions of ESCRT subunits and CSFV proteins, we transfected the plasmids encoding NS3, -NS4B, -NS5A or -NS5B, and examined their subcellular location together with the endogenous ESCRT subunits (HRS, VPS28, VPS25, CHMP2B, CHMP4B, CHMP7, VPS4A and ALIX). As shown in Figs 8A, 8B, S9A and S9B, these ESCRT subunits were co-localized with corresponding nonstructural (NS) proteins, and located in the ER rather than Golgi. These results suggest that these ESCRT subunits are involved in the formation of VCR on ER.

**Fig 9:**
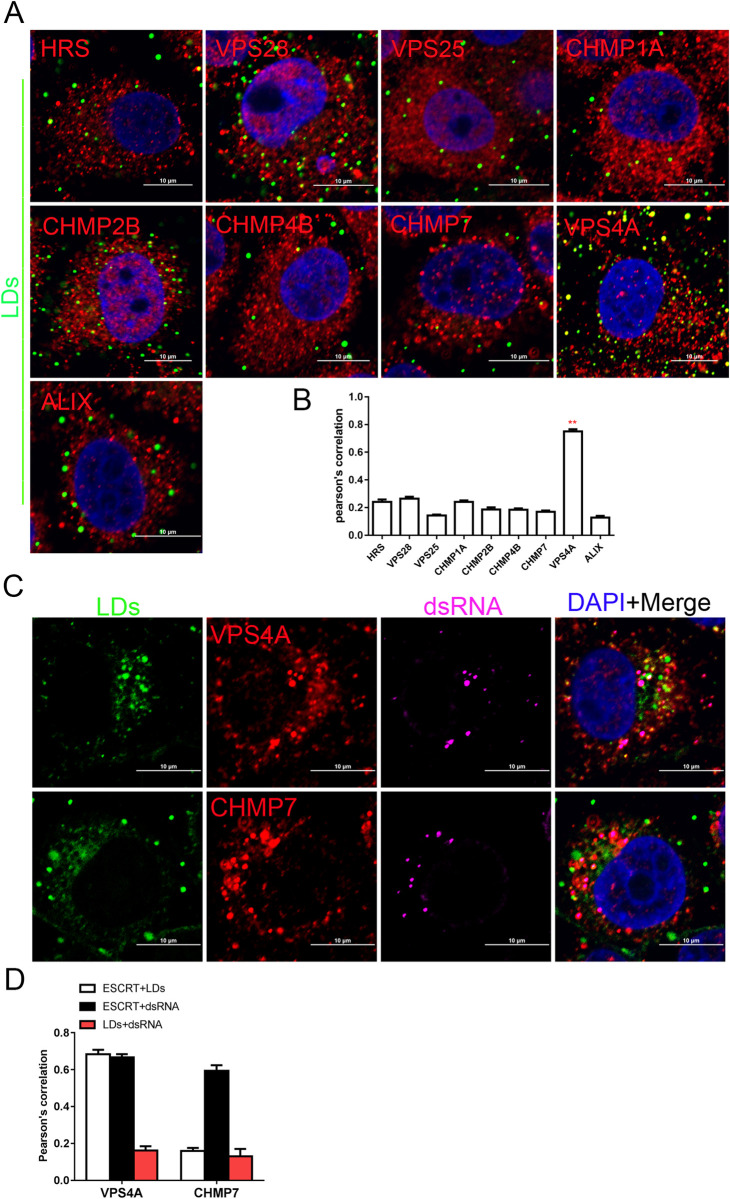
VPS4A protein is closely related to lipid droplets (LDs). (A) PK-15 cells were infected with CSFV (MOI = 1) for 24 hpi, then fixed and subjected to immunofluorescent by using rabbit anti-ESCRTs antibody (red) and dyeing with BODIPY (green). The nuclei were stained with DAPI. Bars = 10 μm. These data are representative of three independent experiments. (B) The colocalization analysis of ESCRT subunits and LDs was expressed as Pearson’s correlation coefficient. Results are represented as the mean + SD of data from three independent experiments. **, P < 0.01. (C) PK-15 cells were infected with CSFV (MOI = 1) for 24 hpi, then fixed and subjected to immunofluorescent by using rabbit anti-VPS4A/CHMP7 antibody (red), mouse anti-dsRNA (purple), and dyeing with BODIPY (green). The nuclei were stained with DAPI. Bars = 10 μm. These data are representative of three independent experiments. (D) The colocalization analysis of VPS4A or CHMP7, dsRNA, and LDs were expressed as Pearson’s correlation coefficient. The white column indicates the co-localization of the ESCRT subunits and LDs, the black column indicates the co-localization of the ESCRT subunits and dsRNA, and the red column indicates the co-localization of the LDs and dsRNA. Results are represented as the mean + SD of data from three independent experiments.

Lipid droplets (LDs), highly dynamic organelles involved in energy homeostasis and membrane transport [38,39], are generated from ER membrane and play important roles in Flavivirus replication [40,41]. To determine the spatial relationships between LDs and ESCRT subunits, ESCRTs accumulated in the ER membrane domains of CSFV-infected cells was stained with BODIPY lipid dye before fixation and staining with various antibodies. These experiments revealed that LDs only co-localized with VPS4A, but not with other ESCRT subunits, such as HRS, VPS28, VPS25, CHMP1A, CHMP2B, CHMP4B, CHMP7 and ALIX (Fig 9A and 9B). Interestingly, we observed that VPS4A, but not CHMP7, separately overlapped with dsRNA and LDs signals, however, dsRNA did not overlap with LDs, indicating that VPS4A was involved not only in VRC but also in LDs (Fig 9C and 9D). These results demonstrate that LDs participating in CSFV infection are associated with the related ESCRT subunits.

### Assembly of ESCRT subunits upon CSFV infection

**Fig 10:**
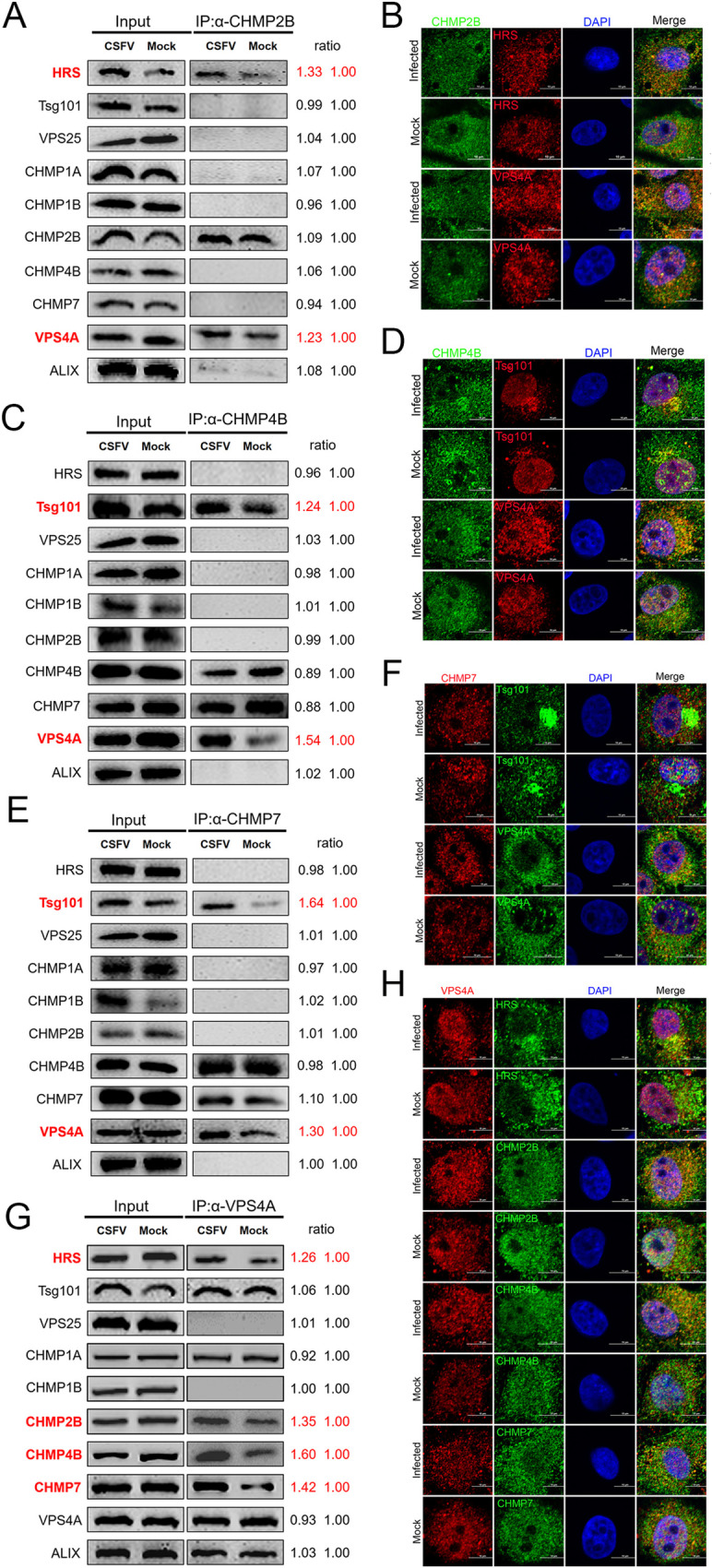
Assembly between major subunit proteins of ESCRT after CSFV infection. (A, C, E and G) PK-15 cells were infected with CSFV (MOI = 1) for 24 hpi and harvested for immunoprecipitation by using rabbit anti-CHMP2B (A); -CHMP4B (C); -CHMP7 (E) antibody, or mouse anti-VPS4A antibody (G), and the whole-cell lysates were subjected to Western blotting by using the antibodies against HRS, Tsg101, VPS25, CHMP1A, CHMP1B, CHMP2B, CHMP4B, CHMP7, VPS4A and ALIX. These data are representative of three independent experiments. (B, D, F and H) PK-15 cells were infected with CSFV (MOI = 1) for 24 hpi, then fixed and subjected to immunofluorescent by using rabbit anti-CHMP2B (B); -CHMP4B (D) antibody (green) and mouse anti-HRS/VPS4A/Tsg101 antibody (red); or mouse anti-CHMP7 antibody (F) (red) and rabbit anti-Tsg101/VPS4A antibody (green); or mouse anti-VPS4A antibody (H) (red) and rabbit anti-HRS/CHMP2B/CHMP4B/CHMP7 antibody (green). The nuclei were stained with DAPI. Bars = 10 μm. These data are representative of three independent experiments.

To confirm the recruitment of the ESCRT subunits upon CSFV infection, we performed co-immunoprecipitation experiments using antibodies for each ESCRT subunit and compared the amounts of other co-precipitated ESCRT subunits in mock and CSFV-infected cells. As shown in Figs 10A and S10A, HRS and VPS4A were co-immunoprecipitated with CHMP2B, and the amounts of HRS and VPS4A in the CHMP2B’s co-precipitates were significantly increased after CSFV infection. In addition, the colocalization between CHMP2B and HRS or VPS4A were verified by immunofluorescence experiments (Fig 10B). Pearson’s correlation analysis also confirmed these colocalizations (S10B Fig). These results indicate that there was an ESCRT complexes existed containing HRS, VPS4A and CHMP2B during CSFV infection. Furthermore, it was found that CHMP4B and CHMP7 increased their interaction with Tsg101 and VPS4A after CSFV infection (Figs 10C, 10D, 10E, 10F, S10C, S10D, S10E and S10F), indicating that another ESCRT complexes formed containing CHMP4B, CHMP7, Tsg101 and VPS4A. Interestingly, the interaction between VPS4A and HRS, CHMP2B, CHMP4B and CHMP7 were also enhanced after CSFV infection (Figs 10G, 10H, S10G and S10H). Altogether, the results suggest that the ESCRT complexes containing specific ESCRT subunits, mainly HRS, Tsg101, CHMP2B, CHMP4B, CHMP7 and VPS4A, are shaped around VCR on the ER membrane and may form vesicles that are required for CSFV genome replication.

## Discussion

**Fig 11:**
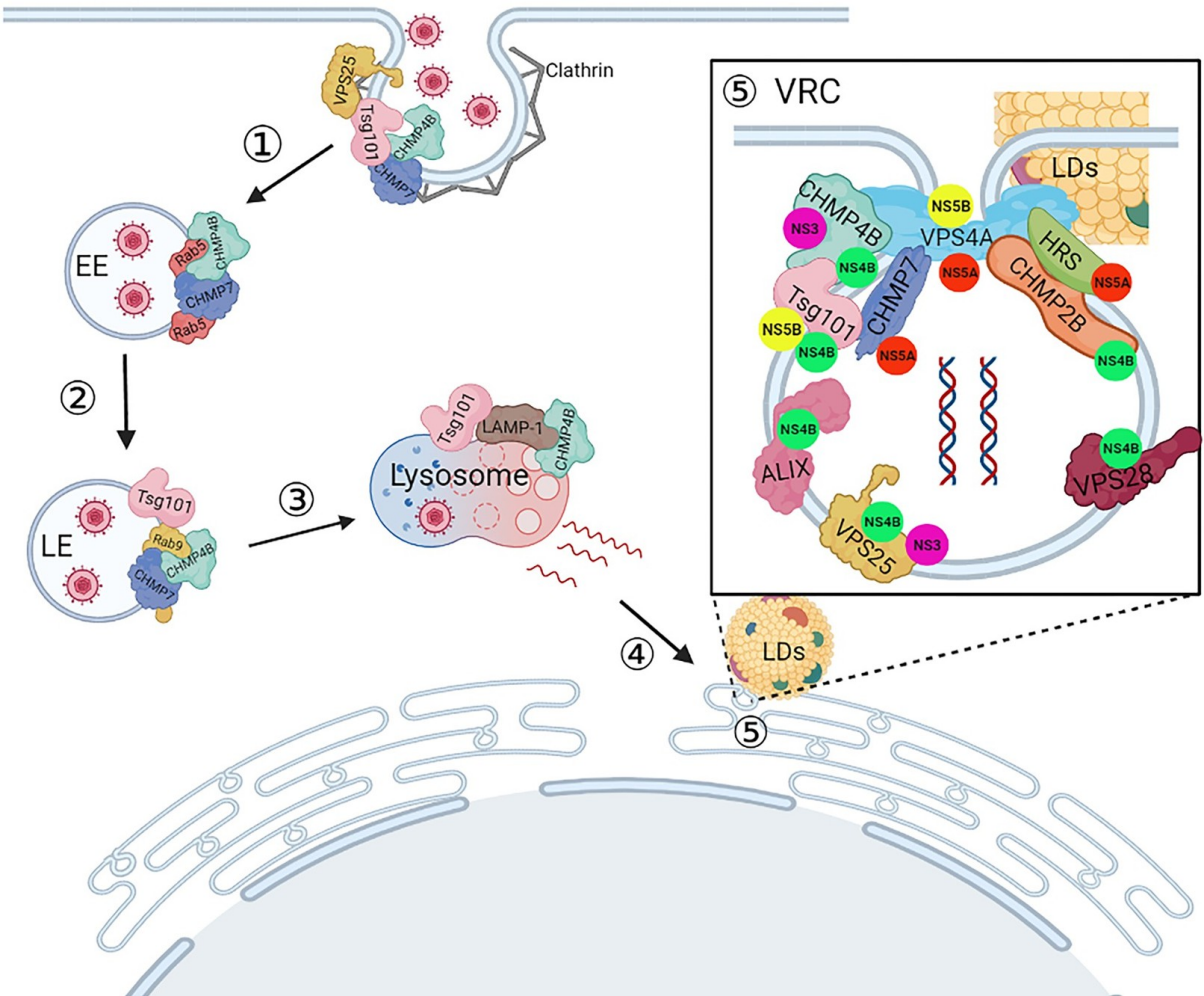
Schematic model depicting CSFV life cycle and the role of ESCRT proteins in PK-15 cells. (1) Clathrin interacted with Tsg101, VPS25, CHMP4B and CHMP7 proteins upon CSFV entry, then transported to the early endosomes. (2) In the early endosomes, CHMP4B and CHMP7 proteins interacted with Rab5 protein and assist the transport of CSFV to the late endosomes. (3) In the late endosomes, Tsg101, CHMP4B, and CHMP7 proteins interacted with Rab9 protein and transport CSFV to the lysosomes. (4) Later, in the lysosomes, the Tsg101 and CHMP4B proteins interacted of with LAMP-1 leads to uncoating and released nucleic acid, then transported to the endoplasmic reticulum area. (5) Finally, ESCRT proteins interacted with nonstructural proteins of CSFV and formed a virus replication complex (VRC) in the ER lumens for viral genome replication. Finally, LDs were closed to the VRC and connected by VPS4A protein.

In this study, we demonstrated that the ESCRT pathway is involved not only in CSFV entry and genome replication but also in virus particle formation (budding). Besides describing the role of Tsg101 during CSFV infection in the previous study [26], here, we systematically provided a new schematic about ESCRT subunits in the CSFV life cycle, as shown in Fig 11. 1) Tsg101, VPS25, CHMP4B and CHMP7 proteins play essential roles in viral trafficking and Clathrin-mediated viral entry. 2) CHMP4B and CHMP7 interact with Rab5 to transport CSFV virions from early endosomes to late endosomes. 3) In the late endosomes, Tsg101, CHMP4B and CHMP7 interact with Rab9 to mediate transport of CSFV to the lysosomes for a productive infection. 4) Tsg101 and CHMP4B associate with LAMP-1 in the lysosomes, probably leading to uncoating and release of nucleic acid for genome replication in the endoplasmic reticulum area. 5) During CSFV genome replication, the ESCRT subunits interact with nonstructural proteins of CSFV and dsRNA to form a virus replication complex (VRC) in the ER lumens, in which the lipid droplets (LDs) may serve as the energy platform [42].

ESCRT system usually participates in multivesicular body (MVB) biogenesis, through endocytosis, ubiquitin-labeled membrane proteins are transported to the endosomal membrane and released into the endosomal cavity to form intraluminal vesicles (ILV) with the assistance of ESCRT [43,44]. These endosomes are called multivesicular bodies (MVBs) and eventually fused with lysosomes to degrade the proteins inside, many of which are membrane-bound receptors [45–47]. A few studies have revealed ESCRT-I (Tsg101) and ESCRT-III (CHMP5/6) are involved in the transport process of Kaposi’s sarcoma-associated herpesvirus (KSHV) invasion, in which Tsg101 forms a complex with CHMP5/6 and helps the virus transport from early endosomes (Rab5) to late endosomes (Rab7) and finally to the nucleus for replication [31,48]. Similarly, we also found that Tsg101 assists in transporting CSFV from early endosomes to late endosomes through microfilaments with the help of motor protein dynein [26,49]. Meanwhile, Tsg101 regulates virus replication through forming the replication complex together with CSFV NS4B/5B nonstructural proteins and dsRNA in the endoplasmic reticulum. In addition, a recent study showed that the dominant-negative mutant (DN) of VPS4 protein significantly inhibits the invasion of autographa californica nuclear polyhedrosis virus (AcMNPV) [50], suggesting VPS4 is involved in virus endocytosis. In this study, we demonstrated that VPS25, CHMP4B and CHMP7 played important roles in the early invasion step of CSFV. Further, VPS25, CHMP4B and CHMP7 interacted with Clathrin to mediate CSFV entry. CHMP4B and CHMP7 were first found to recruit into the early endosomes by interacting with Rab5, then transferred to the late endosomes by interacting with Rab9. In the late endosomes, CHMP4B was associated with LAMP-1 and transported to the lysosomes. Our study comprehensively characterizes how the ESCRT machine regulates Pestivirus entry and trafficking through intracellular endosomes.

During viral infection, the mini-organelles associated with the endoplasmic reticulum are shaped and act as viral RNA replication factories. Replication complex (RC) is composed of multiple viral, host factors and replication organelles (ROs), such as the ER. A universal feature of (+) RNA viruses is that they multiply their RNA on intracellular membranes, requiring vesiculation or other membrane rearrangements [8,51]. Cellular membranes, together with some necessary host factors, usually serve as a scaffold to localize viral components and protect viral RNA from cellular defense mechanisms [52,53]. Remarkably, VRC requires extensive subcellular membrane deformation, in which ESCRTs proteins play important roles [54,55]. Moreover, ESCRT-I and ESCRT-III have been shown to exert on the membrane deformation and invagination, leading to the formation of ILV and multivesicular endosomes [28,56]. Our study found that HRS, VPS28, VPS25, CHMP2B/4B/7, VPS4A and ALIX are relevant to CSFV VRC assembly and facilitate viral replication by interacting with nonstructural proteins. Besides, CSFV nonstructural proteins were assembled into viral replication complexes through binding with host proteins and viral RNA. Coincidentally, the tomato bushy stunt virus (TBSV) nonstructural proteins induce curvature in the ER membranes, forming spherical invaginations into the ER lumen with a pore connecting them to the cytoplasm [57]. Therefore, we speculate that these ESCRT subunits could contribute to the formation of vesicle-like structures-globules, similar to the members of Flaviviridae family replication organelles described previously. VPS4 complex is necessary for the formation of VRC organelles. The interaction between VPS4 subunits and viral nonstructural proteins may be involved in the stability of globule neck structure, allowing continuous ATP synthesis and exporting of newly produced ESCRT machinery [58]. Importantly, the membranous globule structure VRC isolates all replication factors, protecting fragile viral RNA from degradation by host ribonuclease [55]. Herein, the assembly of membranous VRC, relevant to ESCRT subunits, is essential in the replication of (+) RNA viruses.

Previously, a large number of studies have shown that envelope viruses complete the budding and release of virus particles relying on the host cell ESCRT system [17,59]. So far, ESCRT regulating virus budding and releasing are mainly described in retrovirus infection. Japanese encephalitis virus (JEV) and dengue virus (DENV) are members of Flavivirus, their budding from host cells depends on the ESCRT system [58], which is consistent with our data in CSFV. Recent studies have shown that ESCRT-III subunits CHMP6 and CHMP4 help viral genome replication and viral particle assembly by directly remodeling the ER membrane [60]. In the present study, we showed that HRS, Tsg101, VPS28, EAP20, CHMP1B, CHMP2B, CHMP4B, CHMP7, VPS4A and ALIX played unique roles in CSFV budding. The molecular mechanisms will be clarified in the future.

In conclusion, the work highlights the new roles of ESCRT subunits in the entry and replication mechanism of CSFV, enriching the frame in the life cycles of Flavivirus. In addition, these findings illuminate that the positive-strand RNA viruses can integrate functions of viral and host factors and remodel membranes, which may provide alternative ways to control the viruses.

## Materials and methods

### Virus, cells, and plasmids

Classical Swine Fever virus (CSFV) Shimen strain (GenBank accession number: AF092448) was used in this study as described previously [23,26]. Porcine Kidney cells (PK-15) were maintained in Dulbecco’s modified essential medium (DMEM, GIBCO, Invitrogen, USA) supplemented with 10% fetal bovine serum (FBS) (GIBCO, Invitrogen, USA), 0.2% NaHCO_3_, 100 μg/mL streptomycin, and 100 IU/mL penicillin (GIBCO, Invitrogen, USA) at 37°C with 5% CO_2_. The pFlag-Core, pFlag-E2, pFlag-NS3, pFlag-NS4B, pFlag-NS5A, pFlag-NS5B plasmids were generated by cloning corresponding CSFV genes into the p3×Flag-CMV-7.1 vector. The pYFP-CHMP DN constructs were kindly provided by Dr. Colin M. Crump [28]. The pRFP-HRS, pGFP-CHMP2B and pGFP-VPS4A constructs were purchased from Addgene Company (Addgene, USA). The authenticity of each construct was confirmed by DNA sequencing.

### Plasmids and siRNA transfections

PK-15 cells were grown to 70% confluence on coverslip dishes and transfected with an indicated plasmid (2.5 μg) using Lipofectamine 3000 (Invitrogen, USA) according to the manufacturer’s instructions. At 6 h post-transfection (hpt), the transfection mixture was replaced with DMEM containing 2% FBS, and cells were incubated for an additional 48 hpt. For RNA knockdown, PK-15 cells were transfected with siRNA using Lipofectamine RNAiMAX (Invitrogen, USA) according to the manufacturer’s instructions. The siRNA duplex and negative control siRNA used in the study were synthesized by Gene Pharma (GenePharma, China). At 48 hpt, cells were infected with CSFV, and virus replication was evaluated by RT-qPCR, Western blotting or confocal fluorescence microscopy at 24 post-infection (hpi). The siRNA sequences targeted these ESCRT subunits in this study were listed in Additional File 1: S1 Table.

### Confocal fluorescence microscopy

In order to visualize the CSFV and ESCRT subunits at early entry stage, PK-15 cells grown on dishes were infected with CSFV (MOI = 10) at 4°C for 1 h rinsed, then shifted to 37°C for indicated time points. After incubation, the monolayers were fixed with 4% PFA in PBS and permeabilized with 0.1% Triton X-100, after fixed and subjected to confocal microscopy by using mouse anti-CSFV E2 antibody (WH303) [23,26], rabbit anti-VPS25/CHMP4B/CHMP7 antibody (Proteintech, USA) or rabbit anti-VPS4A antibody (Abcam, UK). To investigate the colocalizations of ESCRT subunits and endosomes in CSFV endocytosis, PK-15 cells were treated as described above and infected with CSFV (MOI = 10) at 37°C for 6 hpi, after fixed and subjected to confocal microscopy by using mouse anti-VPS25/CHMP7 antibody (Santa-cruz, USA) and rabbit anti-Clathrin antibody (Cell-signaling-technology, USA), rabbit anti-Rabs/LAMP-1 antibody (Abcam, UK). But for visualize the colocalization of CHMP4B and Clathrin, Rabs or LAMP-1, cells were fixed and subjected to confocal microscopy by using rabbit anti-CHMP4B antibody (Proteintech, USA) and mouse anti-Clathrin/Rabs/LAMP-1 antibody (Santa-cruz, USA). Additionally, for the visualization of the co-localization of dsRNA and ESCRT subunits, PK-15 cells were infected with CSFV (MOI = 1) for 24 hpi, then fixed and subjected to confocal microscopy by using rabbit anti-ESCRTs antibody and mouse double-stranded RNA (dsRNA) monoclonal antibody J2 (Scisons, Hungary). Furthermore, to study the co-localizations of endogenous or exogenous ESCRT subunits and CSFV structural or nonstructural proteins, PK-15 cells were transfected with pFlag plasmids (pFlag-Core, -E2, -NS3, -NS4B, -NS5A, or -NS5B) for 48 hpt, then fixed and subjected to confocal microscopy by using rabbit anti-ESCRTs antibody and mouse anti-Flag antibody (Invitrogen, USA). Moreover, for visualization of the ESCRT subunits, CSFV non-structural proteins and ER or Golgi, PK-15 cells were transfected with pFlag plasmids (pFlag-NS3, -NS4B, -NS5A, or -NS5B) for 48 hpt, then fixed and subjected to confocal microscopy by using goat anti-Flag antibody (Abcam, UK), mouse anti-HRS/VPS28/VPS25/CHMP7/VPS4A/ALIX antibody and endoplasmic reticulum (ER) were stained with rabbit anti-Calnexin antibody (Abcam, UK), and Golgi apparatus were stained with rabbit anti-GM130 antibody (Abcam, UK), respectively. But for visualization of CHMP2B/CHMP4B, ER or Golgi and CSFV non-structural proteins, cells were fixed and subjected to confocal microscopy by using goat anti-Flag antibody (Abcam, UK), rabbit anti-CHMP2B/CHMP4B antibody (Proteintech, USA) and mouse anti-Calnexin antibody (Proteintech, USA) or mouse anti-GM130 antibody (Abcam, UK), respectively. In order to study the co-localizations between LDs and ESCRT subunits, PK-15 cells were infected with CSFV (MOI = 1) for 24 hpi, then fixed and subjected to confocal microscopy by using rabbit anti-ESCRTs antibody and incubated with BODIPY (Invitrogen, USA), but for visualization of LDs, CHMP7、VPS4A, and dsRNA, cells were fixed and subjected to confocal microscopy by using mouse double-stranded RNA (dsRNA) monoclonal antibody J2 and rabbit anti-CHMP7 antibody, rabbit anti-VPS4A antibody and incubated with BODIPY. Finally, to study the assembly between ESCRT subunits after CSFV infection, PK-15 cells were infected with CSFV (MOI = 1) for 24 hpi, then fixed and subjected to confocal microscopy by using rabbit anti-CHMP2B/CHMP4B antibody and mouse anti-HRS/VPS4A/Tsg101 antibody; or mouse anti-CHMP7 antibody and rabbit anti-Tsg101/VPS4A antibody; or mouse anti-VPS4A antibody and rabbit anti-HRS/CHMP2B/CHMP4B/CHMP7 antibody. The colocalization coefficients were calculated using professional quantitative colocalization analysis software (Nikon A1, Nikon, Japan) included with a Nikon A1 confocal microscope and expressed as a Pearson’s correlation coefficient.

### Co-immunoprecipitation (Co-IP) and Western blotting

Firstly, in order to study the interaction between ESCRT subunits and endosomal proteins during CSFV entry. PK-15 cells were infected with CSFV (MOI = 10) at 37°C for 6 hpi, then cells were lysed in NP-40 lysis buffer (50mM Tris-HCl, 150mM NaCl, 1%NP40, 1mM EDTA, 1mM PMSF, 1mM NaF, 1mM Na_3_O_4_, pH = 7.4) for 30 minutes at 4°C. Lysates were cleared by centrifugation at 1,000 × g for 10 min at 4°C. The 20% aliquot of the supernatant (whole cell lysate) was removed from all samples for later use. The 80% remaining lysate was incubated with 0.5 μg of the appropriate control IgG and 20 μl of a Protein A/G PLUS-Agarose slurry (Santa-cruz, USA) for 4 h at 4°C with rotation. Agarose beads were removed by centrifugation at 1,000 × g for 5 minutes at 4°C. The lysates were then incubated with mouse anti-VPS25 antibody (Santa-cruz, USA) or rabbit anti-CHMP4B/CHMP7 antibody (Proteintech, USA). Protein A/G PLUS-Agarose slurry was added to each sample and incubated continued for another 2 h under the same conditions. The agarose beads were collected by centrifugation and washed with NP-40 lysis buffer at least three times, then resuspended in 2 × SDS loading buffer for SDS-PAGE and Western blotting. Immunoprecipitations and whole-cell lysates were harvested and subjected to Western blotting by using mouse anti-ALIX/Tsg101/VPS4A/CHMP7 antibody (Santa-cruz, USA), rabbit anti-VPS25/CHMP4B antibody (Proteintech, USA) and rabbit anti-Clathrin/Rabs/LAMP-1 antibody (Cell-signaling-technology, USA). In addition, to investigate the interaction between ESCRT subunits and CSFV non-structural proteins, HEK-293T cells were transfected with pFlag plasmids (pFlag-NS3, -NS4B, -NS5A, -NS5B, -Core, -E2 and vector) respectively. At 48 hpt, cells were lysed in NP-40 lysis buffer for the same experiment operation as above. The lysates were then treated with mouse anti-Flag antibody (Invitrogen, USA) or mouse anti-HRS/VPS28/VPS25/VPS4A antibody (Santa-cruz, USA), rabbit anti-CHMP2B/CHMP4B/ALIX antibody (Cell-signaling-technology, USA), rabbit anti-CHMP7 antibody (Proteintech, USA), then incubated with rotation for 4–6 h at 4°C. And through a series of experimental steps as described above, and finally Western blotting were used for testing. Immunoprecipitations and whole-cell lysates were harvested and subjected to Western blotting by using the indicated antibodies, β-actin was used as a loading control. Finally, to examine the interaction between the main subunit proteins of ESCRT after CSFV infection, PK-15 cells were infected with CSFV (MOI = 1) for 24 hpi and harvested for Co-IP assay. To determine levels of indicated proteins, the corresponding protein/β-actin or CSFV/mock quantity is used to calculate the grayscale using ImageJ 7.0 software.

### Crud replication complex (CRC) extraction

Cells were infected with or without CSFV (MOI of 1 and 0.1), collected after 24 hpi, washed with pre-cooled PBS, and centrifuged at 800 × g for 10 min. The precipitate was suspended in hypotonic buffer, swelled and ground, and centrifuged at 1,000 × g for 10 min. The supernatant was centrifuged at 68,500 × g for 1 h, resuspend to obtain the CRC, and stored at -80°C [61,62]. Finally, ESCRT subunits proteins, GM130, Calnexin and β-actin were detected by using Western blotting, and the CSFV RNA copies were quantified by real-time RT-PCR.

### Cell viability assay

PK-15 cells were seeded in a 96-well plate and treated with different amounts of siRNA for 48 hpt. The cytotoxic effect of the siRNA on PK-15 cells was evaluated by a CCK8 assay. In addition, the reagent’s fluorescence was measured with a fluorescence microplate reader after 4h of incubation at 37°C. No cytotoxicity was observed in cells treated with the indicated concentrations of the siRNA duplexes.

### Electron microscopy

The samples were treated according to a transmission electron microscopy (TEM) protocol. Cells were fixed for 4 h at room temperature in 2.5% glutaraldehyde in 0.1 M phosphate buffer at pH 7.4, washed with double-distilled water, and dehydrated with a graded ethanol series of (30%, 50%, 70%, 80% and 90%). Finally, the cells were embedded in fresh 100% LR white resin using beem capsules. Eighty-nanometer sections were cut on a Leica UCT ultra microtome and collected onto 400-mesh high-transmission grids. Finally, the sample grids were examined with a transmission electron microscope at 80 kV (JEM-100SX TEM; NEC, Tokyo, Japan).

### Statistical analysis

All data were presented as means ± standard deviations (SD) as indicated. In addition, student’s t-test was used to compare the data from pairs of treated and untreated groups. Statistical significance is indicated by asterisks (*, P<0.05; **, P<0.01; ***, P<0.001) in the figures. All statistical analyses and calculations were performed using Prism 6 (Graph Pad Software, Inc, La Jolla, CA).

## Supporting information

S1 Fig ESCRT subunits are involved in CSFV infection. (A) PK-15 cells were transfected with all siRNA, respectively, then using the CCK8 assay to assess cell viabilities upon all siRNA duplexes. These data are presented as the mean + SD of data from three independent experiments. (B) PK-15 cells were transfected with siESCRTs or siCtrl and then inoculated with CSFV (MOI = 1), and the cells were harvested for RT-qPCR at 1 hpi. These data are presented as the mean + SD of data from three independent experiments. *, P< 0.05; **, P <0.01. (C) PK-15 cells were transfected with siESCRTs or siCtrl and then inoculated with CSFV (MOI = 0.01), then harvested the whole cell cultures at 24 hpi for RT-qPCR. These data are presented as the mean + SD of data from three independent experiments. *, P< 0.05; **, P <0.01. (D) PK-15 cells were transfected with siESCRTs or siCtrl and then inoculated with CSFV (MOI = 0.01), at 24 hpi, the cell supernatant were harvested and used for infected new PK-15 cells, and the new cells were then harvested 24 hpi for RT-qPCR. These data are presented as the mean + SD of data from three independent experiments. *, P< 0.05; **, P <0.01. (E and F) The ratios of ESCRT/β-actin and Npro/β-actin in Fig 1B were analyzed of grayscale analysis through image J software. These data are presented as the mean + SD of data from three independent experiments. *, P< 0.05; **, P <0.01. (G) PK-15 cells were transfected with siESCRTs or siCtrl and then inoculated with CSFV (MOI = 1). At 24 hpi, the cells were fixed and subjected to immunofluorescent by using rabbit anti-CHMP2B/CHMP4B/CHMP7/VPS4A antibody (green) and mouse anti-E2 antibody (red). The nuclei were stained with DAPI. Bars = 10 μm. These data are representative of three independent experiments.
(TIF)

S2 Fig VPS25/CHMP4B/CHMP7 are involved in CSFV endocytosis. (A) The colocalization analysis of CSFV and VPS25/CHMP4B/CHMP7/VPS4A in Fig 2A to 2D were indicated by Pearson’s correlation coefficient, respectively. Results are represented as the mean + SD of data from three independent experiments. (B, C, and D) The Western blotting results of immunoprecipitation in Fig 2E to 2G were analysis through image J software, respectively. These data are presented as the mean + SD of data from three independent experiments. **, P <0.01.
(TIF)

S3 Fig VPS25 protein interacts with Clathrin to mediate CSFV endocytosis. (A) PK-15 cells were infected with CSFV or not (MOI = 10) at 37°C for 6 hpi, after fixed and subjected to immunofluorescent by using mouse anti-VPS25 antibody (red) and rabbit anti-Clathrin/Rabs/LAMP-1(green). The nuclei were stained with DAPI. Bars = 10 μm. These data are representative of three independent experiments. (B) The colocalization analysis was indicated by Pearson’s correlation coefficient, measured for individual cells. Results are represented as the mean + SD of data from three independent experiments. ***, P <0.001.
(TIF)

S4 Fig CHMP4B protein delivers CSFV from early endosomes to late endosomes and lysosomes after endocytosis. (A) PK-15 cells were infected with CSFV or not (MOI = 10) at 37°C for 6 hpi, after fixed and subjected to immunofluorescent by using rabbit anti-CHMP4B antibody (red) and mouse anti-Clathrin/Rabs/LAMP-1 antibody (green). The nuclei were stained with DAPI. Bars = 10 μm. These data are representative of three independent experiments. (B) The colocalization analysis was indicated by Pearson’s correlation coefficient, measured for individual cells. Results are represented as the mean + SD of data from three independent experiments. ***, P <0.001.
(TIF)

S5 Fig CHMP7 protein delivers CSFV from early endosomes to late endosomes after endocytosis. (A) PK-15 cells were infected with CSFV or not (MOI = 10) at 37°C for 6 hpi, after fixed and subjected to immunofluorescent by using mouse anti-CHMP7 antibody (red) and rabbit anti-Clathrin/Rabs/LAMP-1 antibody (green). The nuclei were stained with DAPI. Bars = 10 μm. These data are representative of three independent experiments. (B) The colocalization analysis was indicated by Pearson’s correlation coefficient, measured for individual cells. Results are represented as the mean + SD of data from three independent experiments. ***, P <0.001.
(TIF)

S6 Fig Exogenous HRS protein interacts with CSFV proteins. (A) PK-15 cells were co-transfected with RFP-tagged HRS and indicated plasmids (pFlag-Core, -E2, -NS3, -NS4B, -NS5A, -NS5B) for 48 hpt, then fixed and subjected to immunofluorescent by using mouse anti-Flag antibody (green). The nuclei were stained with DAPI. Bars = 10 μm. These data are representative of three independent experiments. (B) The colocalization analysis was indicated by Pearson’s correlation coefficient, measured for individual cells. Results are represented as the mean + SD of data from three independent experiments. **, P <0.01.
(TIF)

S7 Fig Exogenous CHMP2B protein interacts with CSFV proteins. (A) PK-15 cells were co-transfected with GFP-tagged CHMP2B and indicated plasmids (pFlag-Core, -E2, -NS3, -NS4B, -NS5A, -NS5B) for 48 hpt, then fixed and subjected to immunofluorescent by using mouse anti-Flag antibody (red). The nuclei were stained with DAPI. Bars = 10 μm. These data are representative of three independent experiments. (B) The colocalization analysis was indicated by Pearson’s correlation coefficient, measured for individual cells. Results are represented as the mean + SD of data from three independent experiments. **, P <0.01.
(TIF)

S8 Fig Exogenous VPS4A protein interacts with CSFV proteins. (A) PK-15 cells co-transfected with GFP-tagged VPS4A and indicated plasmids (pFlag-Core, -E2, -NS3, -NS4B, -NS5A, -NS5B) for 48 hpt, then fixed and subjected to immunofluorescent by using mouse anti-Flag antibody (red). The nuclei were stained with DAPI. Bars = 10 μm. These data are representative of three independent experiments. (B) The colocalization analysis was indicated by Pearson’s correlation coefficient, measured for individual cells. Results are represented as the mean + SD of data from three independent experiments. **, P <0.01.
(TIF)

S9 Fig Endogenous ESCRT subunits did not locate in Golgi. (A) PK-15 cells were transfected with indicated plasmids (pFlag-NS3, -NS4B, -NS5A, -NS5B) for 48 hpt, then fixed for immunofluorescent by using mouse anti-HRS/VPS28/VPS25/CHMP7/VPS4A/ALIX antibody (green), goat anti-Flag antibody (red) and rabbit anti-GM130 antibody (purple); or rabbit anti-CHMP2B/CHMP4B antibody (green), goat anti-Flag antibody (red) and mouse anti-GM130 antibody (purple). The nuclei were stained with DAPI. Bars = 10 μm. These data are representative of three independent experiments. (B) The colocalization coefficient of ESCRTs, NS (nonstructural proteins) and Golgi was indicated by Pearson’s correlation coefficient. The white column indicates the co-localization of the ESCRT subunits and Golgi, the black column indicates the co-localization of the ESCRT subunits and nonstructural proteins, and the red column indicates the co-localization of the Golgi and nonstructural proteins. Results are represented as the mean + SD of data from three independent experiments.
(TIF)

S10 Fig The interaction between the major subunit proteins of ESCRT after CSFV infection. (A, C, E and G) The Western blotting results of immunoprecipitation in Fig 10A, 10C, 10E and 10G were analysis through image J software, respectively. These data are presented as the mean + SD of data from three independent experiments. *, P< 0.05; **, P <0.01. (B, D, F and H) The colocalization analysis between ESCRT subunits in Fig 10B, 10D, 10F and 10H was expressed as Pearson’s correlation coefficient. Results are represented as the mean + SD of data from three independent experiments.
(TIF)

S1 Table siRNA duplxes used in this study. (XLSX)
