# Supplementary figures and images for: Cellular ESCRT components are recruited to regulate the endocytic trafficking and RNA replication compartment assembly during classical swine fever virus infection
Source: PLoS Pathog. 2022 Feb 4;18(2):e1010294. doi: 10.1371/journal.ppat.1010294 (PMC8849529; doi:10.1371/journal.ppat.1010294)

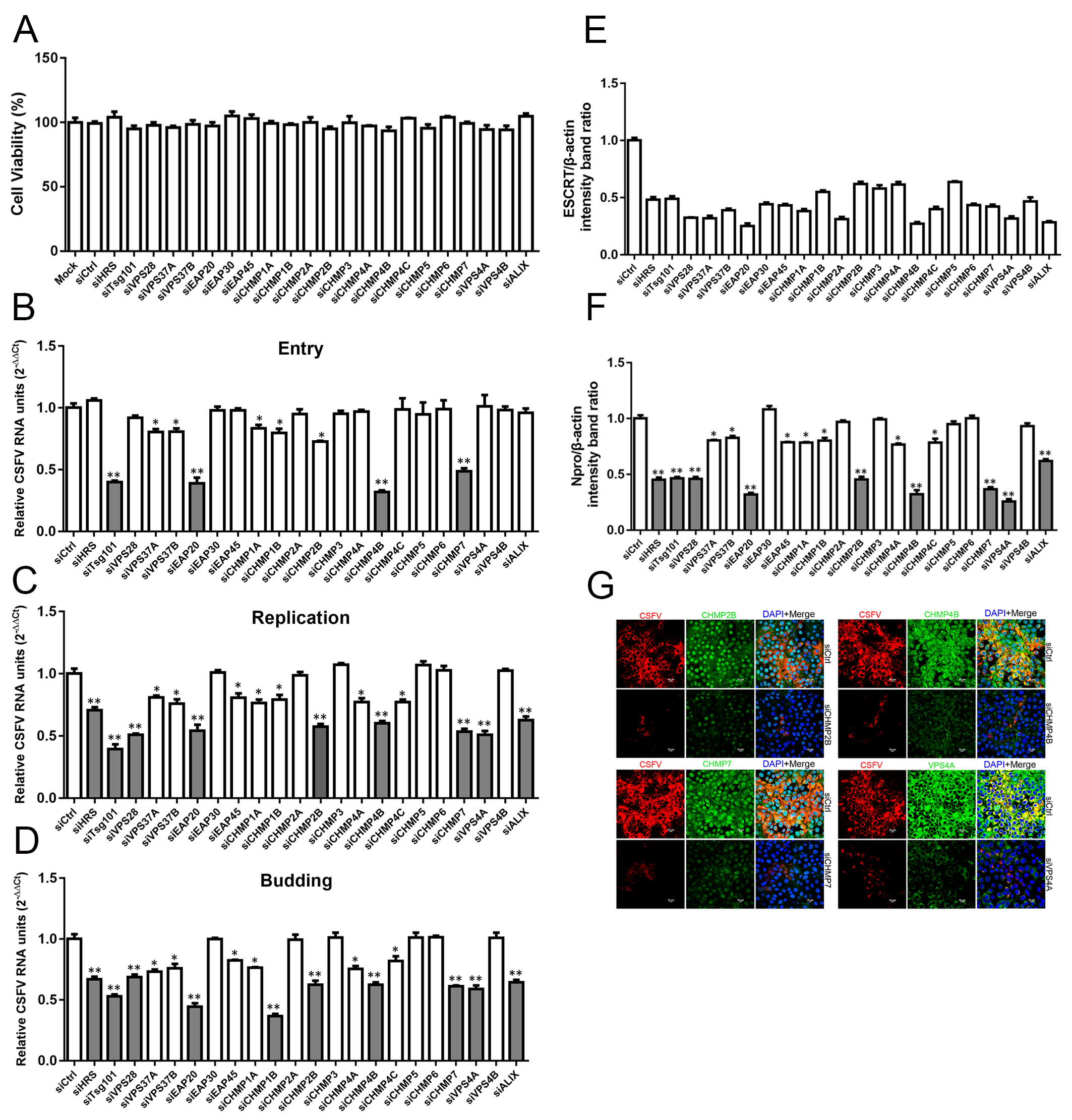

Supplement: S1 Fig — (A) PK-15 cells were transfected with all siRNA, respectively, then using the CCK8 assay to assess cell viabilities upon all siRNA duplexes. These data are presented as the mean + SD of data from three independent experiments. (B) PK-15 cells were transfected with siESCRTs or siCtrl and then inoculated with CSFV (MOI = 1), and the cells were harvested for RT-qPCR at 1 hpi. These data are presented as the mean + SD of data from three independent experiments. *, P< 0.05; **, P <0.01. (C) PK-15 cells were transfected with siESCRTs or siCtrl and then inoculated with CSFV (MOI = 0.01), then harvested the whole cell cultures at 24 hpi for RT-qPCR. These data are presented as the mean + SD of data from three independent experiments. *, P< 0.05; **, P <0.01. (D) PK-15 cells were transfected with siESCRTs or siCtrl and then inoculated with CSFV (MOI = 0.01), at 24 hpi, the cell supernatant were harvested and used for infected new PK-15 cells, and the new cells were then harvested 24 hpi for RT-qPCR. These data are presented as the mean + SD of data from three independent experiments. *, P< 0.05; **, P <0.01. (E and F) The ratios of ESCRT/β-actin and Npro/β-actin in Fig 1B were analyzed of grayscale analysis through image J software. These data are presented as the mean + SD of data from three independent experiments. *, P< 0.05; **, P <0.01. (G) PK-15 cells were transfected with siESCRTs or siCtrl and then inoculated with CSFV (MOI = 1). At 24 hpi, the cells were fixed and subjected to immunofluorescent by using rabbit anti-CHMP2B/CHMP4B/CHMP7/VPS4A antibody (green) and mouse anti-E2 antibody (red). The nuclei were stained with DAPI. Bars = 10 μm. These data are representative of three independent experiments. (TIF) [file ppat.1010294.s001.tif]

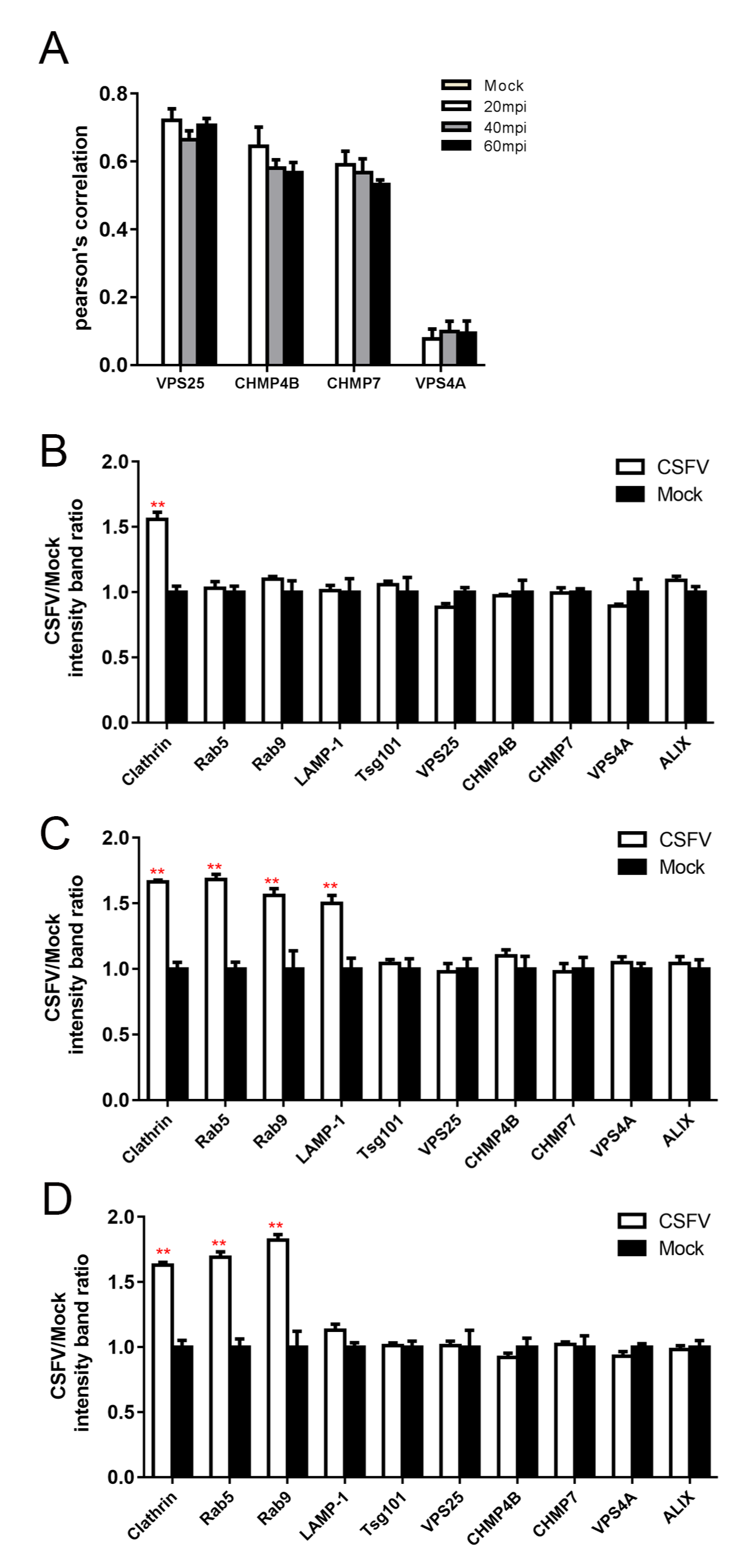

Supplement: S2 Fig — (A) The colocalization analysis of CSFV and VPS25/CHMP4B/CHMP7/VPS4A in Fig 2A to 2D were indicated by Pearson’s correlation coefficient, respectively. Results are represented as the mean + SD of data from three independent experiments. (B, C, and D) The Western blotting results of immunoprecipitation in Fig 2E to 2G were analysis through image J software, respectively. These data are presented as the mean + SD of data from three independent experiments. **, P <0.01. (TIF) [file ppat.1010294.s002.tif]

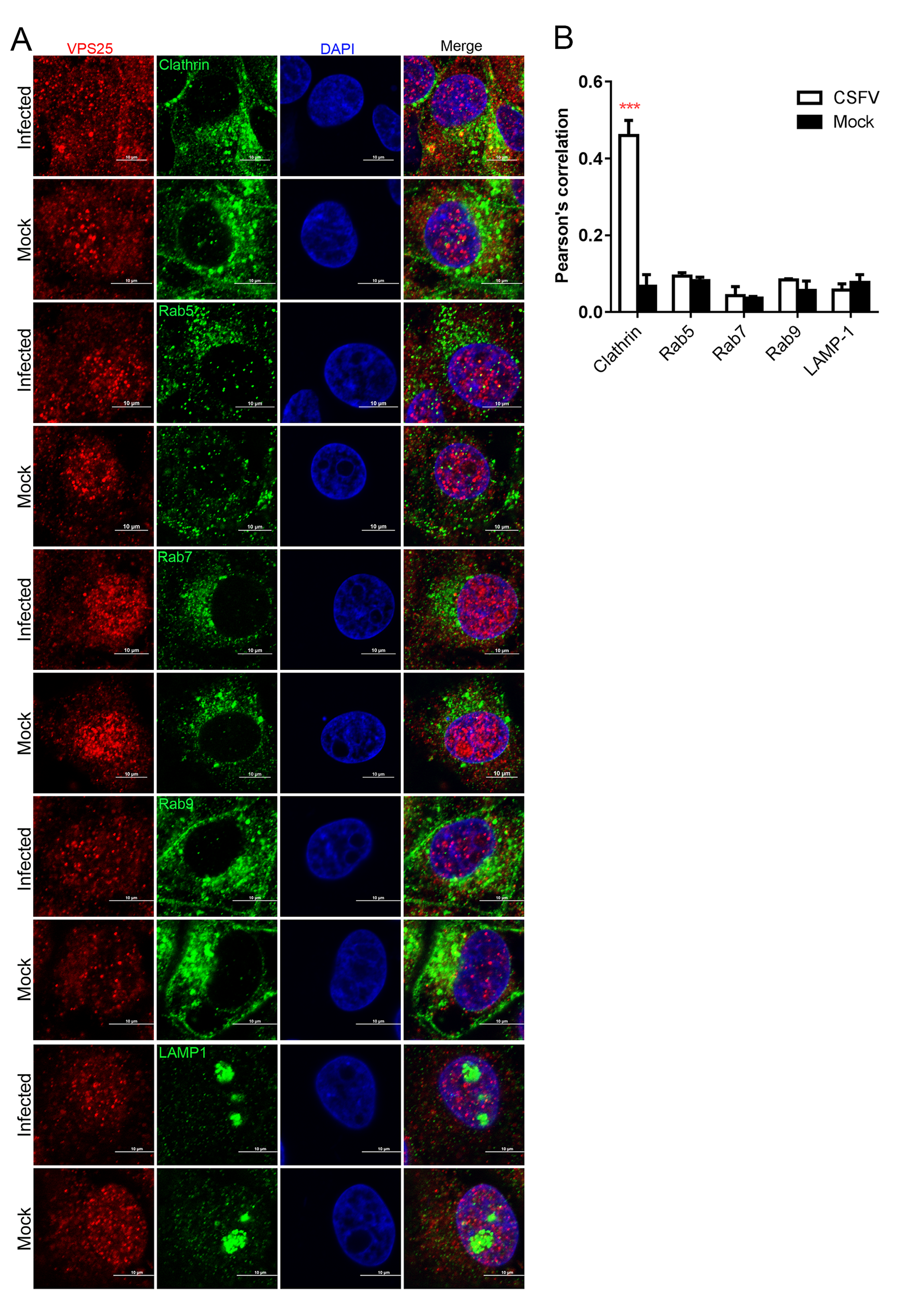

Supplement: S3 Fig — (A) PK-15 cells were infected with CSFV or not (MOI = 10) at 37°C for 6 hpi, after fixed and subjected to immunofluorescent by using mouse anti-VPS25 antibody (red) and rabbit anti-Clathrin/Rabs/LAMP-1(green). The nuclei were stained with DAPI. Bars = 10 μm. These data are representative of three independent experiments. (B) The colocalization analysis was indicated by Pearson’s correlation coefficient, measured for individual cells. Results are represented as the mean + SD of data from three independent experiments. ***, P <0.001. (TIF) [file ppat.1010294.s003.tif]

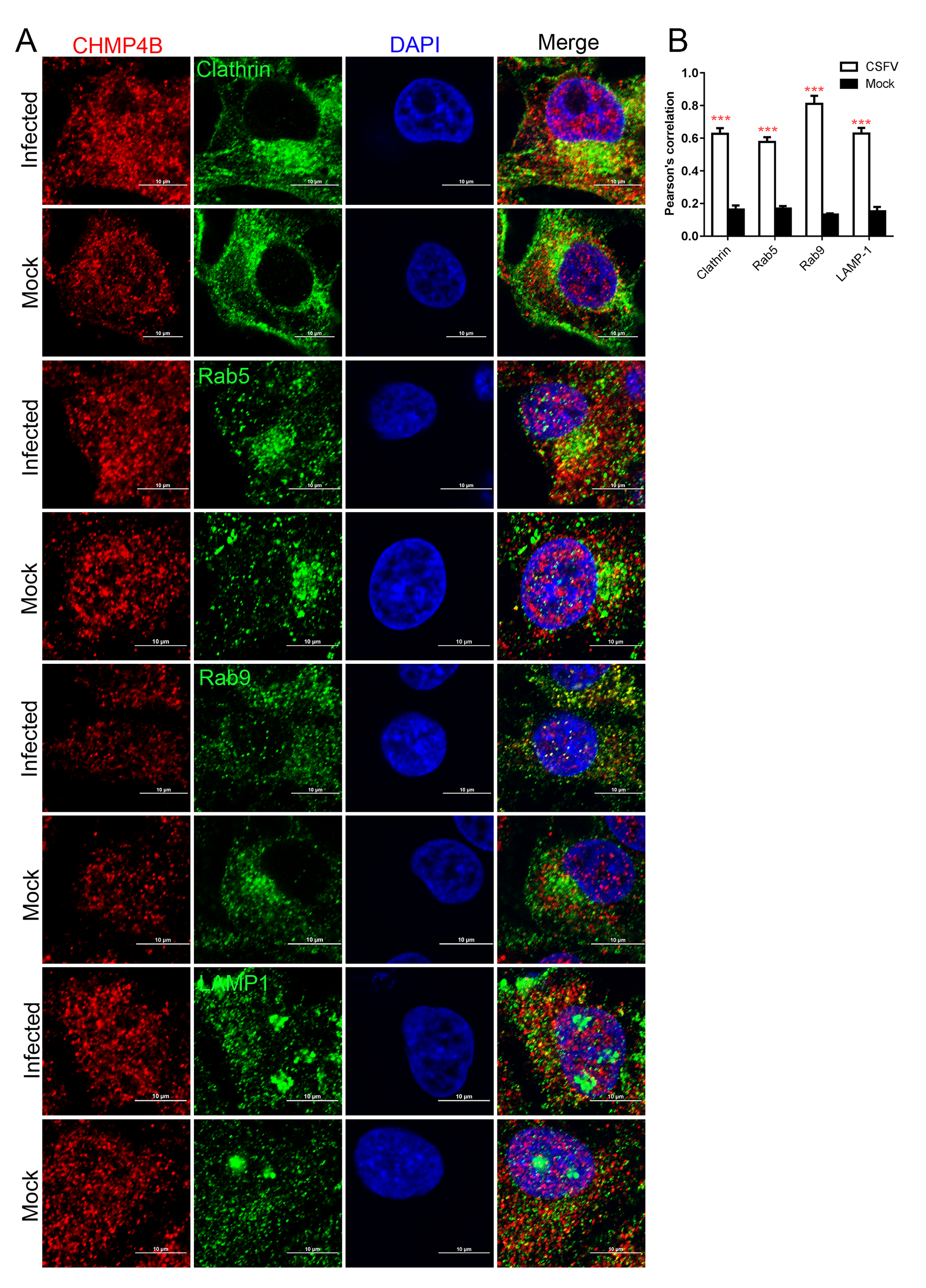

Supplement: S4 Fig — (A) PK-15 cells were infected with CSFV or not (MOI = 10) at 37°C for 6 hpi, after fixed and subjected to immunofluorescent by using rabbit anti-CHMP4B antibody (red) and mouse anti-Clathrin/Rabs/LAMP-1 antibody (green). The nuclei were stained with DAPI. Bars = 10 μm. These data are representative of three independent experiments. (B) The colocalization analysis was indicated by Pearson’s correlation coefficient, measured for individual cells. Results are represented as the mean + SD of data from three independent experiments. ***, P <0.001. (TIF) [file ppat.1010294.s004.tif]

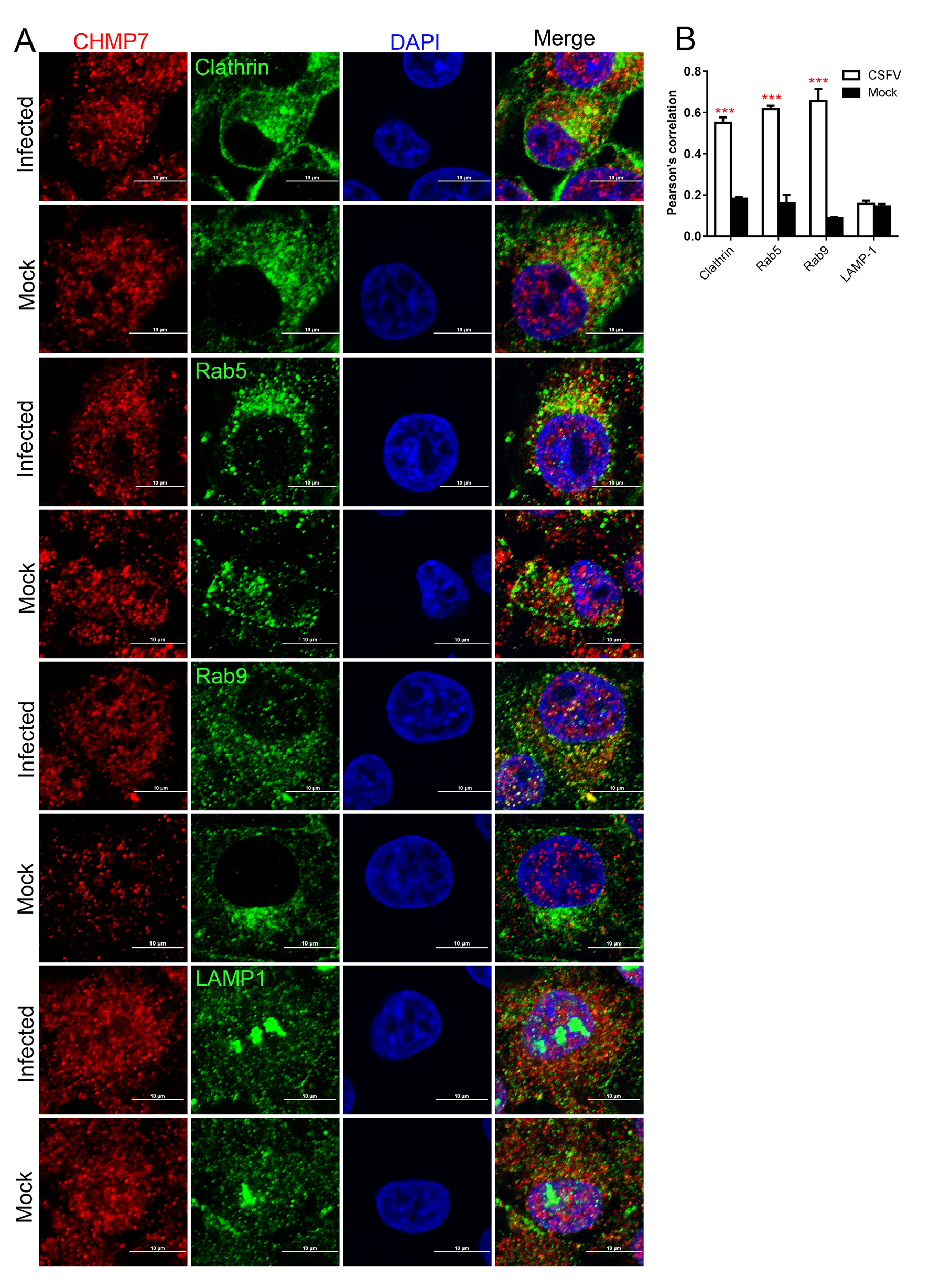

Supplement: S5 Fig — (A) PK-15 cells were infected with CSFV or not (MOI = 10) at 37°C for 6 hpi, after fixed and subjected to immunofluorescent by using mouse anti-CHMP7 antibody (red) and rabbit anti-Clathrin/Rabs/LAMP-1 antibody (green). The nuclei were stained with DAPI. Bars = 10 μm. These data are representative of three independent experiments. (B) The colocalization analysis was indicated by Pearson’s correlation coefficient, measured for individual cells. Results are represented as the mean + SD of data from three independent experiments. ***, P <0.001. (TIF) [file ppat.1010294.s005.tif]

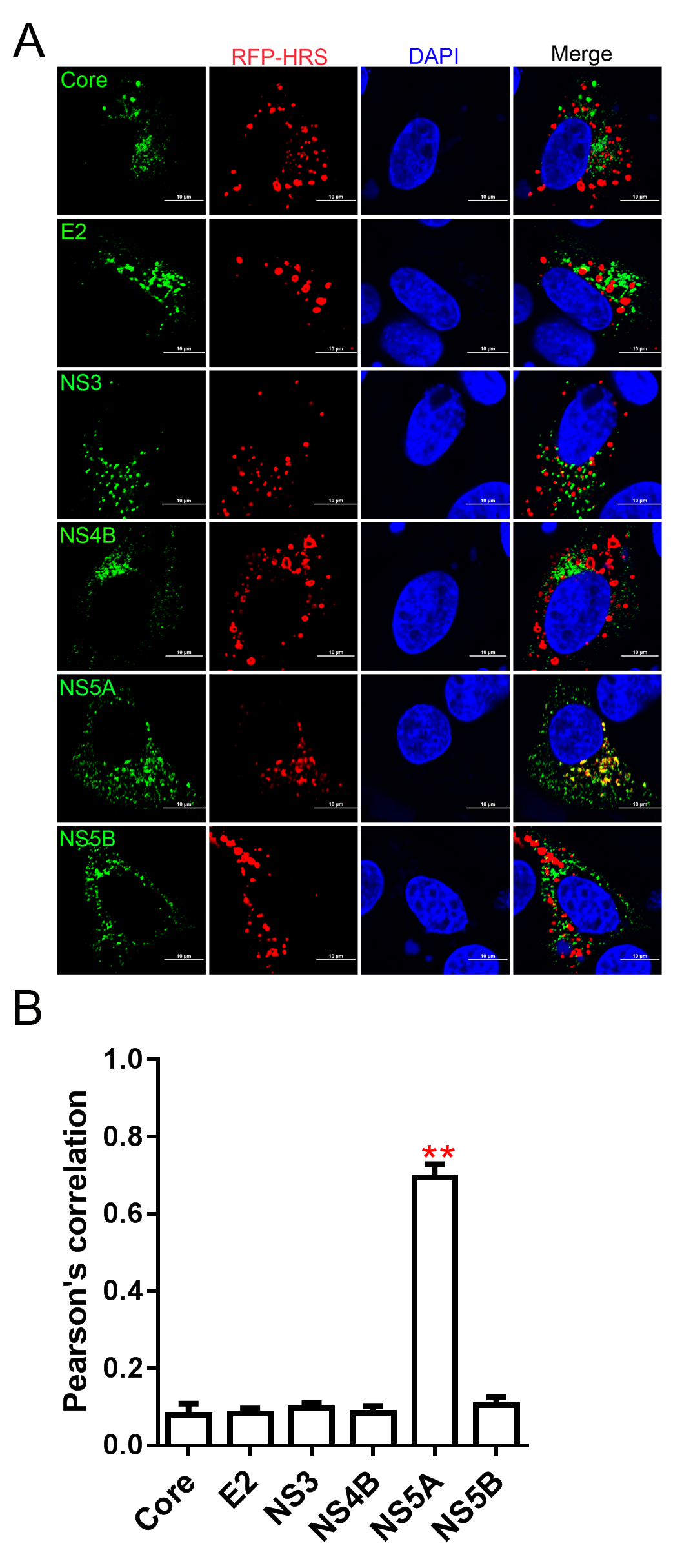

Supplement: S6 Fig — (A) PK-15 cells were co-transfected with RFP-tagged HRS and indicated plasmids (pFlag-Core, -E2, -NS3, -NS4B, -NS5A, -NS5B) for 48 hpt, then fixed and subjected to immunofluorescent by using mouse anti-Flag antibody (green). The nuclei were stained with DAPI. Bars = 10 μm. These data are representative of three independent experiments. (B) The colocalization analysis was indicated by Pearson’s correlation coefficient, measured for individual cells. Results are represented as the mean + SD of data from three independent experiments. **, P <0.01. (TIF) [file ppat.1010294.s006.tif]

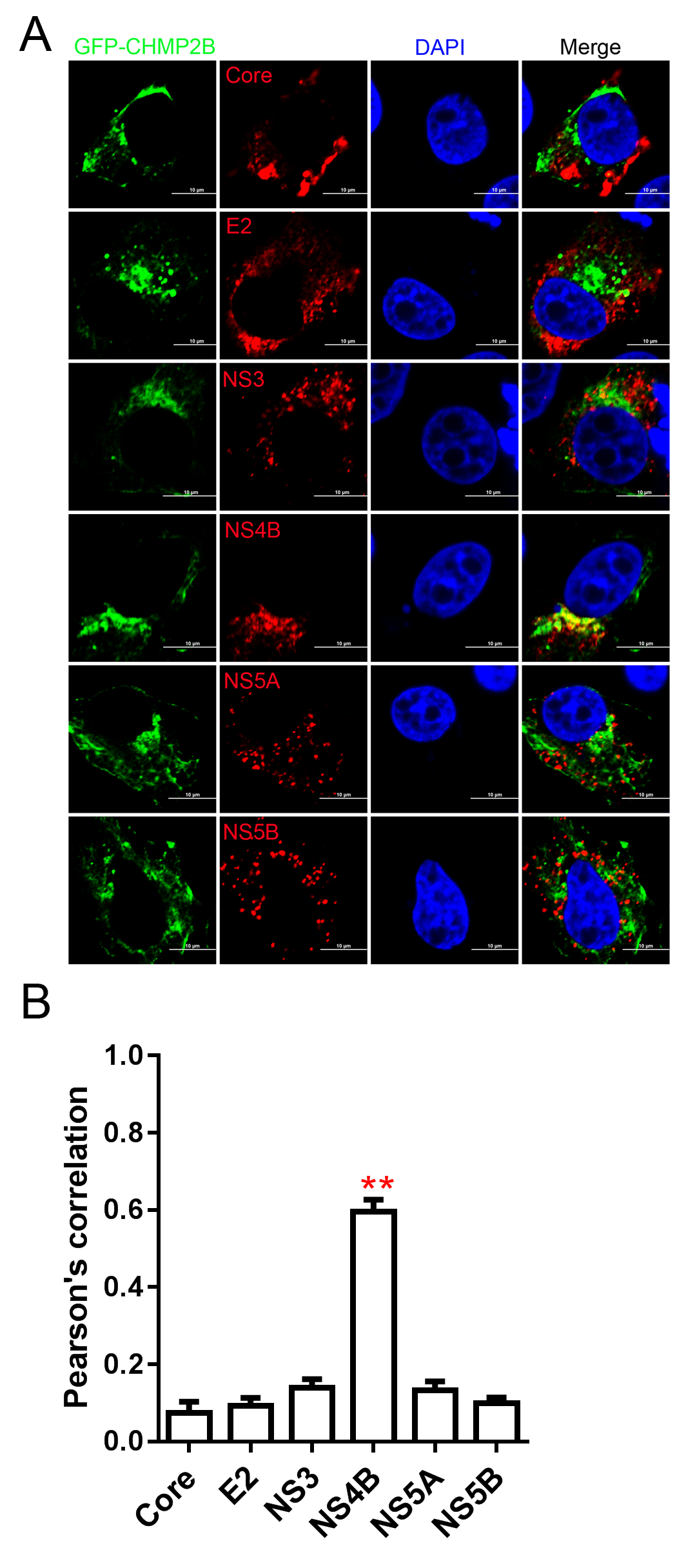

Supplement: S7 Fig — (A) PK-15 cells were co-transfected with GFP-tagged CHMP2B and indicated plasmids (pFlag-Core, -E2, -NS3, -NS4B, -NS5A, -NS5B) for 48 hpt, then fixed and subjected to immunofluorescent by using mouse anti-Flag antibody (red). The nuclei were stained with DAPI. Bars = 10 μm. These data are representative of three independent experiments. (B) The colocalization analysis was indicated by Pearson’s correlation coefficient, measured for individual cells. Results are represented as the mean + SD of data from three independent experiments. **, P <0.01. (TIF) [file ppat.1010294.s007.tif]

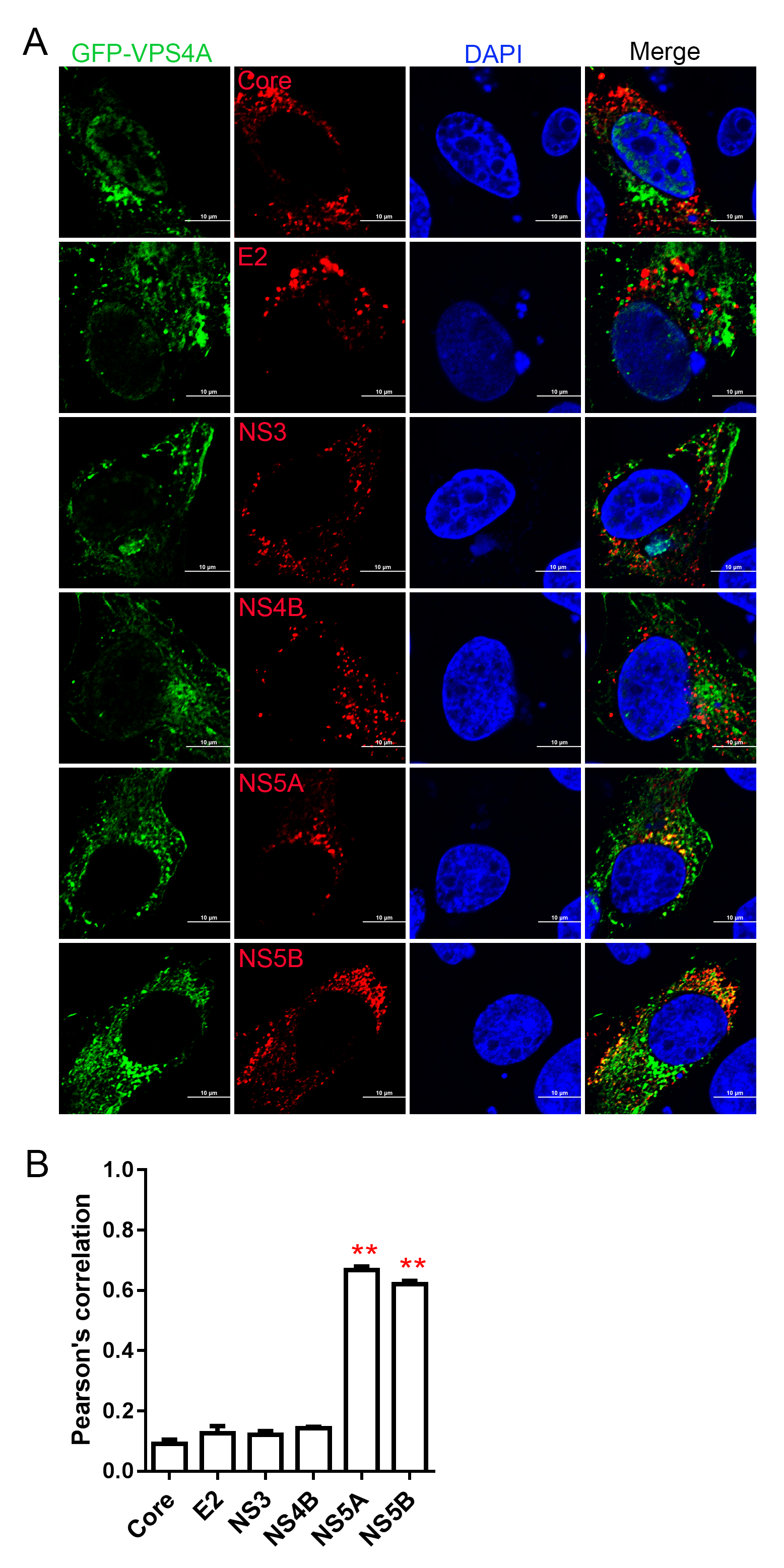

Supplement: S8 Fig — (A) PK-15 cells co-transfected with GFP-tagged VPS4A and indicated plasmids (pFlag-Core, -E2, -NS3, -NS4B, -NS5A, -NS5B) for 48 hpt, then fixed and subjected to immunofluorescent by using mouse anti-Flag antibody (red). The nuclei were stained with DAPI. Bars = 10 μm. These data are representative of three independent experiments. (B) The colocalization analysis was indicated by Pearson’s correlation coefficient, measured for individual cells. Results are represented as the mean + SD of data from three independent experiments. **, P <0.01. (TIF) [file ppat.1010294.s008.tif]

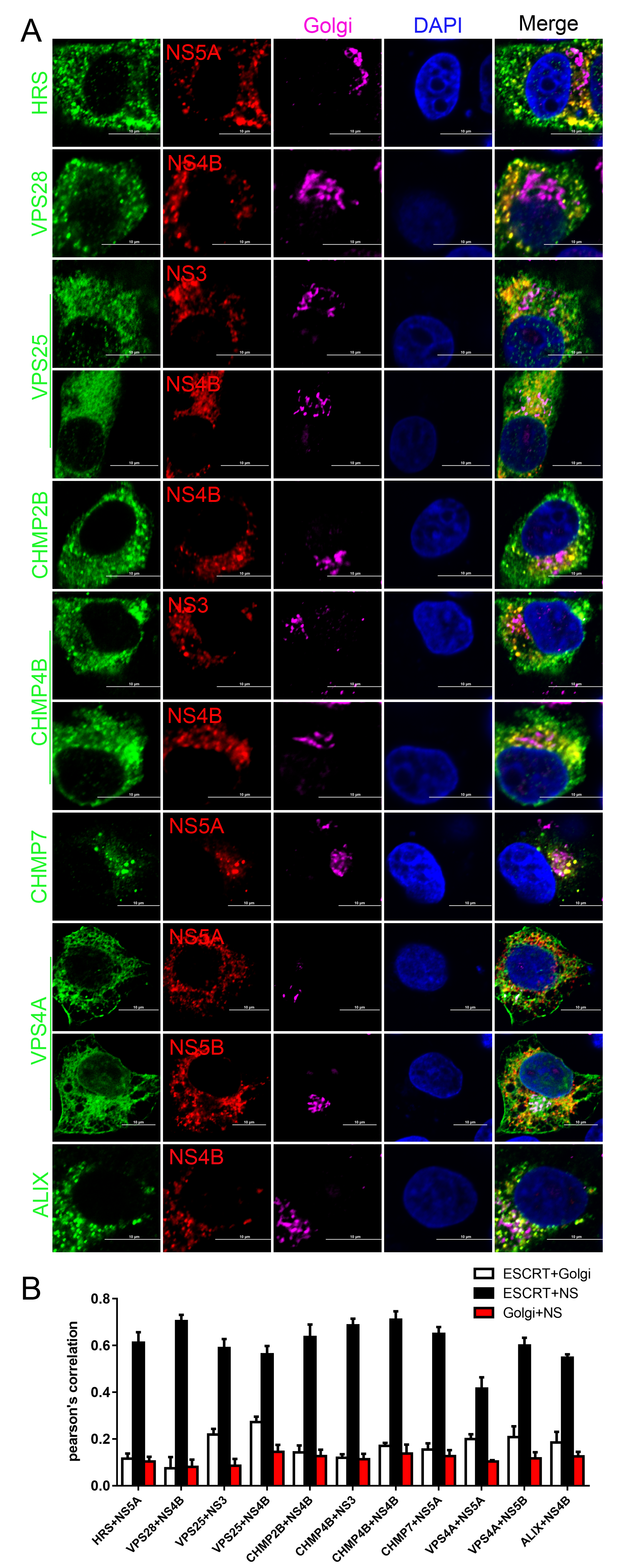

Supplement: S9 Fig — (A) PK-15 cells were transfected with indicated plasmids (pFlag-NS3, -NS4B, -NS5A, -NS5B) for 48 hpt, then fixed for immunofluorescent by using mouse anti-HRS/VPS28/VPS25/CHMP7/VPS4A/ALIX antibody (green), goat anti-Flag antibody (red) and rabbit anti-GM130 antibody (purple); or rabbit anti-CHMP2B/CHMP4B antibody (green), goat anti-Flag antibody (red) and mouse anti-GM130 antibody (purple). The nuclei were stained with DAPI. Bars = 10 μm. These data are representative of three independent experiments. (B) The colocalization coefficient of ESCRTs, NS (nonstructural proteins) and Golgi was indicated by Pearson’s correlation coefficient. The white column indicates the co-localization of the ESCRT subunits and Golgi, the black column indicates the co-localization of the ESCRT subunits and nonstructural proteins, and the red column indicates the co-localization of the Golgi and nonstructural proteins. Results are represented as the mean + SD of data from three independent experiments. (TIF) [file ppat.1010294.s009.tif]

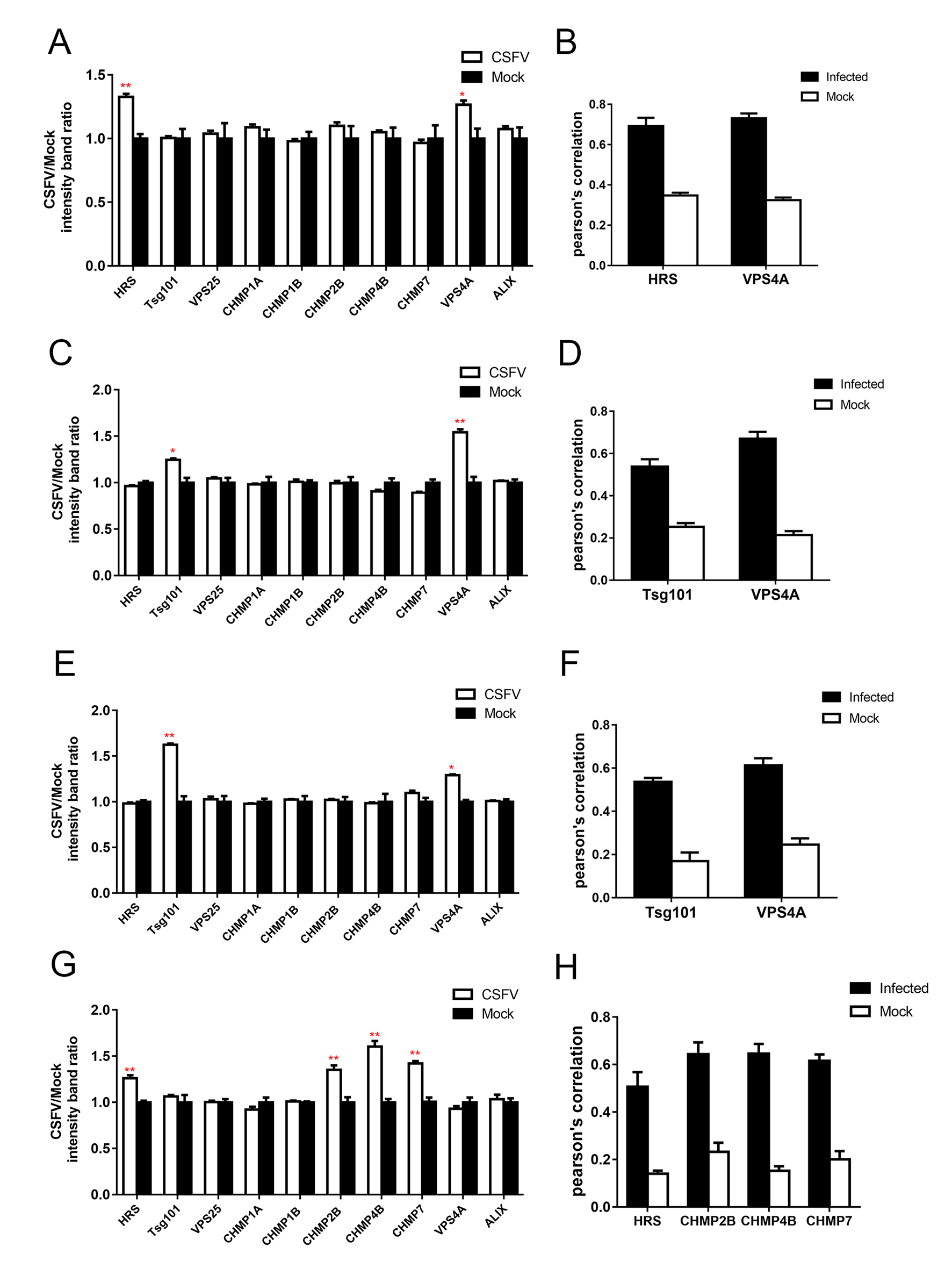

Supplement: S10 Fig — (A, C, E and G) The Western blotting results of immunoprecipitation in Fig 10A, 10C, 10E and 10G were analysis through image J software, respectively. These data are presented as the mean + SD of data from three independent experiments. *, P< 0.05; **, P <0.01. (B, D, F and H) The colocalization analysis between ESCRT subunits in Fig 10B, 10D, 10F and 10H was expressed as Pearson’s correlation coefficient. Results are represented as the mean + SD of data from three independent experiments. (TIF) [file ppat.1010294.s010.tif]
